# Supplementary material for: Maternal and perinatal outcomes after implementation of a more active management in late- and postterm pregnancies in Sweden: A population-based cohort study
Source: PLoS Med. 2025 Jan 16;22(1):e1004504. doi: 10.1371/journal.pmed.1004504 (PMC11737695; doi:10.1371/journal.pmed.1004504)
Supplement: S1 Protocol — (PDF) [file pmed.1004504.s002.pdf]

Outcomes after implementation of a more active management of pregnancies lasting 41 completed weeks or more

Research plan

Background

It has long been known that children born from pregnancies that have lasted longer than 41 completed weeks have a slightly increased risk of perinatal morbidity and mortality (Olesen et al., 2003, Caughey et al., 2005) compared to babies born at 39-40 gestational weeks. Induction of labor (induction) is believed to be able to decrease this risk, but there is no consensus on the optimal gestational age for induction. Results from a randomized trial, the Swedish Postterm Induction Study (SWEPIS) published in 2019, and other studies (Wennerholm et al., 2019, Alkmark et al., 2020, Middleton et al., 2020) have shown that induction at 41+0 gestational weeks (instead of at 42 completed weeks) can reduce the risk of perinatal mortality without increasing the caesarean section rate or maternal morbidity. This has made the Swedish Society for Obstetrics and Gynecology (SFOG) to introduce new national guidelines that recommend induction at 41 completed weeks, or at least an individual plan and aiming at birth or active labour latest at 42 completed weeks (National Clinical Knowledge Support, 2021).

Data reported in the Swedish Pregnancy Register's annual report from 2022 show that all maternity hospitals already in 2020 changed their management so that the induction rate among pregnancies 41+0 weeks or more increased from approximately 35% in 2019 to 50% in 2020 (Petersson et al., 2022). Furthermore, the data showed that the perinatal mortality decreased among pregnancies that lasted 41+0 weeks or more between the period 2017-2019 and 2020-2021 (from 0.17% to 0.10%, RR 0.59, 95% CI 0.42-0.83, p=0.003). The induction rate increased among all hospitals, however, both the increase in induction rate and the induction rate per se varied much among hospitals (inductions between 6% and 80%). The rate of emergency caesarean sections also rose between 2017-2019 and 2020-2021, but it also did so among deliveries before 41 completed weeks.

Aims

We intend to investigate if the new guidelines to offer induction for women with a pregnancy lasting 41+0 weeks or more have reduced the risks of neonatal morbidity and peri/neonatal mortality.

Furthermore, we will investigate whether the new guidelines have led to changes in mode of delivery (rates of caesarean section, vacuum extraction, forceps).

We will analyse the rate of inductions among different maternity hospitals and relate it to outcomes.

Furthermore, we will examine whether a possible risk reduction applies to all groups of women, or whether it differs depending on the individual characteristics of the women (such as primiparous women and/or women with a high BMI).

Analysis plan

Databases:

• Births 2017-2023 from the Swedish Pregnancy Registry

• Linkage to Swedish Neonatal Quality Register (SNQ) for the corresponding year

• Linkage to Statistics Sweden (SCB) and the Register of Total Population (RTB) to obtain date of death.

Primary cohort: All singletons ≥41+0 weeks will be included. Number of non-cephalic presentation and planned C-sections are primarily identified and will be excluded in later analyses.

For sensitivity analyses, data on births between 39+0 and 40+6 gestational weeks are also collected.

Children with severe malformation defined according to the European Surveillance of Congenital Anomalies (EUROCAT 1.5) is primarily included but excluded in a sensitivity analysis.

Two main objectives (analysis and outcomes):

A. **Analysis of management strategies 2017-2022** (up to 2023 if the data can be completed before start of study).
   Calculation of:
   1. Induction rate per hospital-region-major region divided into 2017-2019, 2020-2022–(2023)
   2. Induction rate per gestational length (3-day interval) divided into 2017-2019, 2020-2023
   3. Proportion of women who are currently pregnant at 42+0 weeks
   4. Frequency of births per gestational length (3-day interval) divided by period

   Sensitivity analysis:
   Corresponding analysis is done for pregnancies lasting 39+0 – 40+6 weeks. This is to investigate whether routines have changed for these as well.

Furthermore, deliveries in 2017 will be excluded to investigate whether changes in CTG assessments affected the results (Jonsson et al. 2022).

B. Outcome analyses

Primary outcomes:

1. Perinatal death
2. Composite adverse neonatal outcome: Perinatal death, Apgar 5'<4, admission to neonatal intensive care unit ≥4 days, meconium aspiration (MAS), birth trauma (P10-P15), hypoxic ischemic encephalopathy (HIE) 1-3, neonatal death.
3. Cesarean section rate (planned or emergency + divided)

Secondary outcomes:

Maternal outcomes

1. Childbirth experience (possibly the VAS scale)
2. Ev. self-assessed health at follow-up after childbirth.
3. Bleeding >1000 ml (total, and after caesarean section and vaginal delivery, respectively)
4. Endometritis (total, and after caesarean section and vaginal delivery, respectively)
5. Perineal rupture grade III – IV
6. Hypertension/preeclampsia
7. Instrumental delivery (VE/forceps)

Neonatal outcomes:

The subcomponents of the composite variable:

8. Perinatal death
9. Neonatal death
10. Apgar <4 at 5 minutes
11. Admission to NICU ≥4 days
12. Meconium aspiration
13. Birth trauma (P10-P15)
14. HIE 1
15. HIE 2-3

Other child outcomes

16. Stillbirth
17. SGA, LGA (definition according to Marsál et al., 1996)
18. Macrosomia >4500g
19. Apgar score <7 at 5 minutes

Sensitivity analysis:

Applies to primary outcome: Corresponding outcome analyzes are performed that include pregnancies that lasted 39+0 – 40+6 weeks. This is to investigate whether any outcome changes have also occurred in these pregnancies.

Statistical methods

Analyses will be based on the management routine at each delivery unit – not based on the management of the certain pregnancy. Thus, the exposure is decided by the routine used at the delivery unit at time of birth.

The primary analyses will be made comparing the pregnancy outcome before and after change of management of pregnancies lasting 41 weeks or more.

The initial descriptive analyses will be made to establish how to define the general, but also unit-specific, dates of routine changes (breaking points for the before- and after analyses).

Analyses will be made to investigate possible association between the size of the change (the magnitude of the induction rate increase) and the outcome change before- and after the breaking point.

Analyses will be made, investigating the possible association of unit-specific induction rate and pregnancy outcome.

Sensitivity analyses will be made, investigating the association between changed routines and pregnancy outcomes, stratified by groups. The groups that will be investigated are:

Primiparous-, multiparous without previous CS, and multiparous women with previous CS.

BMI-classes: <18.5, 18.5-24.9, 25-29.9, 30-34.9, and 35+.

Maternal age groups: <25, 25-34, 35-39, 40+

Maternal country of birth: Nordic country, other European, outside Europe.

Statistics

Frequencies will be noted as percent (with 95% CI, using normal approximation when adequate). Possible differences between groups displayed in descriptive tables will be evaluated using Chi2 analyses.

The main analyses will be made using GEE-analyses, considering the dependency within each delivery unit. Adjustment will be made for maternal age, parity, smoking and BMI. However, serious confounding is not expected since each comparison will be made before- and after breaking point for each hospital. The most important analyses will be the stratifications already mentioned.

Power analyses

The main analyses consider three primary outcomes. The main cohort will consist of pregnancies lasting 41+0 weeks or more in Sweden. The estimated numbers are: 2017-2019 N=74 000, and 2020-2022 65 000. In the final analyses, the outcomes at each delivery unit will be compared before and after the breaking points. During the period 2019 the estimated rate of the primary outcomes after pregnancies lasting to 41+0 weeks or more were:

Perinatal death: 0.17%, Composite morbidity: 4.2%, Emergency CS: 12%.

With alpha=0.05 and beta=0.20 the smallest detectable Relative Risk reduction would be:

Perinatal death: We will be able to detect a 33% risk reduction (RR 0.67), or alternatively, a 40% risk increase (RR=1.40).

Child morbidity composite outcome: We will be able to detect a 7% risk reduction (RR 0.93), or alternatively, a 7% risk increase (RR=1.07).

Emergency CS: We will be able to detect a 4% risk reduction (RR 0.96), or alternatively, a 4% risk increase (RR=1.04).

The extremely high power to detect possible associations between changes in management and pregnancy outcome makes it possible to detect possible heterogeneity between the investigated groups (e.g. between parity- or maternal BMI-groups). Thus, we will have good opportunities to identify possible groups that particularly gained (or did not gain) from the changed management.

References

Alkmark M, Keulen JKJ, Kortekaas JC, Bergh C, van Dillen J, Duijnhoven RG, Hagberg H, Mol BW, Molin M, van der Post JAM, Saltvedt S, Wikström AK, Wennerholm UB, de Miranda E. Induction of labour at 41 weeks or expectant management until 42 weeks: A systematic review and an individual participant data meta-analysis of randomised trials. PLoS Med. 2020 Dec 8;17(12):e1003436. doi: 10.1371/journal.pmed.1003436. PMID: 33290410.

Caughey AB, Washington AE, Laros RK Jr. Neonatal complications of term pregnancy: rates by gestational age increase in a continuous, not threshold, fashion. Am J Obstet Gynecol. 2005 Jan;192(1):185-90. doi: 10.1016/j.ajog.2004.06.068. PMID: 15672023.

https://www.nationelltklinisktkunskapsstod.se/globalassets/nkk/media/dokument/kunskapsstod/vardriktlinjer/riktlinje-for-handlaggning-i-graviditetsvecka-41.pdf

https://euplatform.jrc.ec.europa.eu/system/files/public/eurocat/Guide_1.5_Chapter_3.3_June_2 022.pdf

Jonsson M, Söderling J, Ladfors L, Nordström L, Nilsson M, Algovik M, Norman M, Holzmann M. Implementation of a revised classification for intrapartum fetal heart rate monitoring and association to birth outcome: A national cohort study. Acta Obstet Gynecol Scand. 2022 Feb;101(2):183-192. doi: 10.1111/aogs.14296. Epub 2022 Jan 28. PMID: 35092004.

Marsál K, Persson PH, Larsen T, Lilja H, Selbing A, Sultan B. Intrauterine growth curves based on ultrasonically estimated foetal weights. Acta Paediatr. 1996 Jul;85(7):843-8. doi: 10.1111/j.1651-2227.1996.tb14164.x. PMID: 8819552.

Middleton P, Shepherd E, Morris J, Crowther CA, Gomersall JC. Induction of labour at or beyond 37 weeks' gestation. Cochrane Database Syst Rev. 2020 Jul 15;7(7):CD004945. doi: 10.1002/14651858.CD004945.pub5. PMID: 32666584.

Olesen AW, Westergaard JG, Olsen J. Perinatal and maternal complications related to posterm delivery: a national register-based study, 1978-1993. Am J Obstet Gynecol. 2003 Jul;189(1):222-7. doi: 10.1067/mob.2003.446. PMID: 12861166.

Petersson K, Skogsdal Y, Conner P, Sengpiel V, Storck Lindholm E, Kloow M, Elvander L, Granfors M på uppdrag av Graviditetsregistret. Graviditetsregistrets Årsrapport 2021. 2022-09-22. Graviditetsregistret.se

Wennerholm UB, Saltvedt S, Wessberg A, Alkmark M, Bergh C, Wendel SB, Fadl H, Jonsson M, Ladfors L, Sengpiel V, Wesström J, Wennergren G, Wikström AK, Elden H, Stephansson O, Hagberg H. Induction of labour at 41 weeks versus expectant management and induction of labour at 42 weeks (SWEdish Post-term Induction Study, SWEPIS): multicentre, open label, randomised, superiority trial. BMJ. 2019 Nov 20;367:l6131. doi: 10.1136/bmj.l6131.
