# Supplementary material for: Maternal and perinatal outcomes after implementation of a more active management in late- and postterm pregnancies in Sweden: A population-based cohort study
Source: PLoS Med. 2025 Jan 16;22(1):e1004504. doi: 10.1371/journal.pmed.1004504 (PMC11737695; doi:10.1371/journal.pmed.1004504)
Supplement: S1 Syntax Main Analysis — (Armonk, NY: IBM Corp, USA). (PDF) [file pmed.1004504.s003.pdf]

```
* Encoding: UTF-8.

*Impute Missing Data Values.
DATASET DECLARE Final_41plus_Imputed.
MULTIPLE IMPUTATION BMI R□k
  /IMPUTE METHOD=AUTO NIMPUTATIONS=50 MAXPCTMISSING=NONE
  /MISSINGSUMMARIES NONE
  /IMPUTATIONSUMMARIES MODELS
  /OUTFILE IMPUTATIONS=Final_41plus_Imputed .

DATASET ACTIVATE Final_41plus_Imputed.
* Generalized Linear Models.

SPLIT FILE OFF.
*=============================================================================
=========
* Main analyses, primary outcomes: Analyses using data set with multiple imputations

SORT CASES  BY Imputation_.
SPLIT FILE LAYERED BY Imputation_.

* Generalized Linear Models.
GENLIN PeriNeonatalDeath BY ParaKlass UtbClass (ORDER=ASCENDING) WITH Period Age R□k
BMI
  /MODEL ParaKlass Period Age R□k BMI UtbClass INTERCEPT=YES
 DISTRIBUTION=POISSON LINK=LOG
  /CRITERIA METHOD=FISHER(1) SCALE=1 COVB=ROBUST MAXITERATIONS=100 MAXSTEPHA
G=5
    PCONVERGE=1E-006(ABSOLUTE) SINGULAR=1E-012 ANALYSISTYPE=3(WALD) CILEVEL=95
YPE=WALD
    LIKELIHOOD=FULL
  /MISSING CLASSMISSING=EXCLUDE
  /PRINT CPS DESCRIPTIVES MODELINFO FIT SUMMARY SOLUTION (EXPONENTIATED).

* Generalized Linear Models.
GENLIN Composit BY ParaKlass UtbClass (ORDER=ASCENDING) WITH Period Age R□k BMI
  /MODEL ParaKlass Period Age R□k BMI UtbClass INTERCEPT=YES
 DISTRIBUTION=POISSON LINK=LOG
  /CRITERIA METHOD=FISHER(1) SCALE=1 COVB=ROBUST MAXITERATIONS=100 MAXSTEPHA
G=5
    PCONVERGE=1E-006(ABSOLUTE) SINGULAR=1E-012 ANALYSISTYPE=3(WALD) CILEVEL=95
YPE=WALD
    LIKELIHOOD=FULL
  /MISSING CLASSMISSING=EXCLUDE
  /PRINT CPS DESCRIPTIVES MODELINFO FIT SUMMARY SOLUTION (EXPONENTIATED).


* Generalized Linear Models.
GENLIN Composit2 BY ParaKlass UtbClass (ORDER=ASCENDING) WITH Period Age R□k BMI
  /MODEL ParaKlass Period Age R□k BMI UtbClass INTERCEPT=YES
 DISTRIBUTION=POISSON LINK=LOG
```

```
  /CRITERIA METHOD=FISHER(1) SCALE=1 COVB=ROBUST MAXITERATIONS=100 MAXSTEPHA
G=5
    PCONVERGE=1E-006(ABSOLUTE) SINGULAR=1E-012 ANALYSISTYPE=3(WALD) CILEVEL=95
YPE=WALD
    LIKELIHOOD=FULL
  /MISSING CLASSMISSING=EXCLUDE
  /PRINT CPS DESCRIPTIVES MODELINFO FIT SUMMARY SOLUTION (EXPONENTIATED).



* Generalized Linear Models.
GENLIN AkCS BY ParaKlass UtbClass (ORDER=ASCENDING) WITH Period Age R□k BMI
  /MODEL ParaKlass Period Age R□k BMI UtbClass  INTERCEPT=YES
 DISTRIBUTION=POISSON LINK=LOG
  /CRITERIA METHOD=FISHER(1) SCALE=1 COVB=ROBUST MAXITERATIONS=100 MAXSTEPHA
G=5
    PCONVERGE=1E-006(ABSOLUTE) SINGULAR=1E-012 ANALYSISTYPE=3(WALD) CILEVEL=95
YPE=WALD
    LIKELIHOOD=FULL
  /MISSING CLASSMISSING=EXCLUDE
  /PRINT CPS DESCRIPTIVES MODELINFO FIT SUMMARY SOLUTION (EXPONENTIATED).

SPLIT FILE OFF.


*==============================================================================
==============

DATASET ACTIVATE DataSet1.

*Mean imputation data set
''''''''''
*==============================================================================
=========
* Main, crude analyses, primary outcomes: Analyses using data set with multiple imputations

* Generalized Linear Models.
GENLIN PeriNeonatalDeath BY ParaKlass UtbClass (ORDER=ASCENDING) WITH Period Age R□k
BMI
  /MODEL Period INTERCEPT=YES
 DISTRIBUTION=POISSON LINK=LOG
  /CRITERIA METHOD=FISHER(1) SCALE=1 COVB=ROBUST MAXITERATIONS=100 MAXSTEPHA
G=5
    PCONVERGE=1E-006(ABSOLUTE) SINGULAR=1E-012 ANALYSISTYPE=3(WALD) CILEVEL=95
YPE=WALD
    LIKELIHOOD=FULL
  /MISSING CLASSMISSING=EXCLUDE
  /PRINT CPS DESCRIPTIVES MODELINFO FIT SUMMARY SOLUTION (EXPONENTIATED).

* Generalized Linear Models.
GENLIN Composit BY ParaKlass UtbClass (ORDER=ASCENDING) WITH Period Age R□k BMI
```

```
  /MODEL Period INTERCEPT=YES
 DISTRIBUTION=POISSON LINK=LOG
  /CRITERIA METHOD=FISHER(1) SCALE=1 COVB=ROBUST MAXITERATIONS=100 MAXSTEPHA
G=5
    PCONVERGE=1E-006(ABSOLUTE) SINGULAR=1E-012 ANALYSISTYPE=3(WALD) CILEVEL=95
YPE=WALD
    LIKELIHOOD=FULL
  /MISSING CLASSMISSING=EXCLUDE
  /PRINT CPS DESCRIPTIVES MODELINFO FIT SUMMARY SOLUTION (EXPONENTIATED).


* Generalized Linear Models.
GENLIN Composit2 BY ParaKlass UtbClass (ORDER=ASCENDING) WITH Period Age R□k BMI
 /MODEL Period INTERCEPT=YES
 DISTRIBUTION=POISSON LINK=LOG
  /CRITERIA METHOD=FISHER(1) SCALE=1 COVB=ROBUST MAXITERATIONS=100 MAXSTEPHA
G=5
    PCONVERGE=1E-006(ABSOLUTE) SINGULAR=1E-012 ANALYSISTYPE=3(WALD) CILEVEL=95
YPE=WALD
    LIKELIHOOD=FULL
  /MISSING CLASSMISSING=EXCLUDE
  /PRINT CPS DESCRIPTIVES MODELINFO FIT SUMMARY SOLUTION (EXPONENTIATED).



* Generalized Linear Models.
GENLIN AkCS BY ParaKlass UtbClass (ORDER=ASCENDING) WITH Period Age R□k BMI
/MODEL Period INTERCEPT=YES
 DISTRIBUTION=POISSON LINK=LOG
  /CRITERIA METHOD=FISHER(1) SCALE=1 COVB=ROBUST MAXITERATIONS=100 MAXSTEPHA
G=5
    PCONVERGE=1E-006(ABSOLUTE) SINGULAR=1E-012 ANALYSISTYPE=3(WALD) CILEVEL=95
YPE=WALD
    LIKELIHOOD=FULL
  /MISSING CLASSMISSING=EXCLUDE
  /PRINT CPS DESCRIPTIVES MODELINFO FIT SUMMARY SOLUTION (EXPONENTIATED).


***************
* Generalized Linear Models.
GENLIN PeriNeonatalDeath BY ParaKlass (ORDER=DESCENDING) WITH Period Age BMI_1 R□k_1
  /MODEL Period INTERCEPT=YES
 DISTRIBUTION=POISSON LINK=LOG
  /CRITERIA METHOD=FISHER(1) SCALE=1 COVB=ROBUST MAXITERATIONS=100 MAXSTEPHA
G=5
    PCONVERGE=1E-006(ABSOLUTE) SINGULAR=1E-012 ANALYSISTYPE=3(WALD) CILEVEL=95
YPE=WALD
    LIKELIHOOD=FULL
  /MISSING CLASSMISSING=EXCLUDE
  /PRINT CPS DESCRIPTIVES MODELINFO FIT SUMMARY SOLUTION (EXPONENTIATED).
```

```
*================================================================================
=========
* Main, crude analyses, secondary outcomes: Crude, and Analyses using mean imputations for BMI
and Rok (Smoke)

*Stillbirth
* Generalized Linear Models.
GENLIN Stillbirth BY ParaKlass (ORDER=DESCENDING) WITH Period Age BMI_1 R□k_1
  /MODEL Period INTERCEPT=YES
 DISTRIBUTION=POISSON LINK=LOG
  /CRITERIA METHOD=FISHER(1) SCALE=1 COVB=ROBUST MAXITERATIONS=100 MAXSTEPHA
G=5
    PCONVERGE=1E-006(ABSOLUTE) SINGULAR=1E-012 ANALYSISTYPE=3(WALD) CILEVEL=95
YPE=WALD
    LIKELIHOOD=FULL
  /MISSING CLASSMISSING=EXCLUDE
  /PRINT CPS DESCRIPTIVES MODELINFO FIT SUMMARY SOLUTION (EXPONENTIATED).

* Generalized Linear Models.
GENLIN Stillbirth BY ParaKlass UtbClass (ORDER=DESCENDING) WITH Period Age BMI_1 R□k_1
  /MODEL Period ParaKlass Age BMI_1 R□k_1 UtbClass INTERCEPT=YES
 DISTRIBUTION=POISSON LINK=LOG
  /CRITERIA METHOD=FISHER(1) SCALE=1 COVB=ROBUST MAXITERATIONS=100 MAXSTEPHA
G=5
    PCONVERGE=1E-006(ABSOLUTE) SINGULAR=1E-012 ANALYSISTYPE=3(WALD) CILEVEL=95
YPE=WALD
    LIKELIHOOD=FULL
  /MISSING CLASSMISSING=EXCLUDE
  /PRINT CPS DESCRIPTIVES MODELINFO FIT SUMMARY SOLUTION (EXPONENTIATED).


*Perinatal death
* Generalized Linear Models.
GENLIN PerinatalDeath BY ParaKlass (ORDER=DESCENDING) WITH Period Age BMI_1 R□k_1
  /MODEL Period INTERCEPT=YES
 DISTRIBUTION=POISSON LINK=LOG
  /CRITERIA METHOD=FISHER(1) SCALE=1 COVB=ROBUST MAXITERATIONS=100 MAXSTEPHA
G=5
    PCONVERGE=1E-006(ABSOLUTE) SINGULAR=1E-012 ANALYSISTYPE=3(WALD) CILEVEL=95
YPE=WALD
    LIKELIHOOD=FULL
  /MISSING CLASSMISSING=EXCLUDE
  /PRINT CPS DESCRIPTIVES MODELINFO FIT SUMMARY SOLUTION (EXPONENTIATED).

* Generalized Linear Models.
GENLIN PerinatalDeath BY ParaKlass UtbClass (ORDER=DESCENDING) WITH Period Age BMI_1
R□k_1
  /MODEL Period ParaKlass Age BMI_1 R□k_1 UtbClass INTERCEPT=YES
 DISTRIBUTION=POISSON LINK=LOG
  /CRITERIA METHOD=FISHER(1) SCALE=1 COVB=ROBUST MAXITERATIONS=100 MAXSTEPHA
G=5
```

```
    PCONVERGE=1E-006(ABSOLUTE) SINGULAR=1E-012 ANALYSISTYPE=3(WALD) CILEVEL=95
YPE=WALD
    LIKELIHOOD=FULL
  /MISSING CLASSMISSING=EXCLUDE
  /PRINT CPS DESCRIPTIVES MODELINFO FIT SUMMARY SOLUTION (EXPONENTIATED).

* For outcomes live births only ======================================

USE ALL.
COMPUTE filter_$=(D□d <> 1).
VARIABLE LABELS filter_$ 'D□d <> 1 (FILTER)'.
VALUE LABELS filter_$ 0 'Not Selected' 1 'Selected'.
FORMATS filter_$ (f1.0).
FILTER BY filter_$.
EXECUTE.

* Generalized Linear Models.
GENLIN NeonatalDeath BY ParaKlass (ORDER=DESCENDING) WITH Period Age BMI_1 R□k_1
  /MODEL Period INTERCEPT=YES
 DISTRIBUTION=POISSON LINK=LOG
  /CRITERIA METHOD=FISHER(1) SCALE=1 COVB=ROBUST MAXITERATIONS=100 MAXSTEPHA
G=5
    PCONVERGE=1E-006(ABSOLUTE) SINGULAR=1E-012 ANALYSISTYPE=3(WALD) CILEVEL=95
YPE=WALD
    LIKELIHOOD=FULL
  /MISSING CLASSMISSING=EXCLUDE
  /PRINT CPS DESCRIPTIVES MODELINFO FIT SUMMARY SOLUTION (EXPONENTIATED).

* Generalized Linear Models.
GENLIN NeonatalDeath BY ParaKlass UtbClass (ORDER=DESCENDING) WITH Period Age BMI_1
R□k_1
  /MODEL Period ParaKlass Age BMI_1 R□k_1 UtbClass INTERCEPT=YES
 DISTRIBUTION=POISSON LINK=LOG
  /CRITERIA METHOD=FISHER(1) SCALE=1 COVB=ROBUST MAXITERATIONS=100 MAXSTEPHA
G=5
    PCONVERGE=1E-006(ABSOLUTE) SINGULAR=1E-012 ANALYSISTYPE=3(WALD) CILEVEL=95
YPE=WALD
    LIKELIHOOD=FULL
  /MISSING CLASSMISSING=EXCLUDE
  /PRINT CPS DESCRIPTIVES MODELINFO FIT SUMMARY SOLUTION (EXPONENTIATED).



USE ALL.
COMPUTE filter_$=(D□d <> 1).
VARIABLE LABELS filter_$ 'D□d <> 1 (FILTER)'.
VALUE LABELS filter_$ 0 'Not Selected' 1 'Selected'.
FORMATS filter_$ (f1.0).
FILTER BY filter_$.
EXECUTE.
```

```
* Generalized Linear Models.
GENLIN Apg5lth4 BY ParaKlass (ORDER=DESCENDING) WITH Period Age BMI_1 R□k_1
  /MODEL Period INTERCEPT=YES
 DISTRIBUTION=POISSON LINK=LOG
  /CRITERIA METHOD=FISHER(1) SCALE=1 COVB=ROBUST MAXITERATIONS=100 MAXSTEPHA
G=5
    PCONVERGE=1E-006(ABSOLUTE) SINGULAR=1E-012 ANALYSISTYPE=3(WALD) CILEVEL=95
YPE=WALD
    LIKELIHOOD=FULL
  /MISSING CLASSMISSING=EXCLUDE
  /PRINT CPS DESCRIPTIVES MODELINFO FIT SUMMARY SOLUTION (EXPONENTIATED).

* Generalized Linear Models.
GENLIN Apg5lth4 BY ParaKlass UtbClass (ORDER=DESCENDING) WITH Period Age BMI_1 R□k_1
  /MODEL Period ParaKlass Age BMI_1 R□k_1 UtbClass INTERCEPT=YES
 DISTRIBUTION=POISSON LINK=LOG
  /CRITERIA METHOD=FISHER(1) SCALE=1 COVB=ROBUST MAXITERATIONS=100 MAXSTEPHA
G=5
    PCONVERGE=1E-006(ABSOLUTE) SINGULAR=1E-012 ANALYSISTYPE=3(WALD) CILEVEL=95
YPE=WALD
    LIKELIHOOD=FULL
  /MISSING CLASSMISSING=EXCLUDE
  /PRINT CPS DESCRIPTIVES MODELINFO FIT SUMMARY SOLUTION (EXPONENTIATED).

* Generalized Linear Models.
GENLIN Apg5lth7 BY ParaKlass (ORDER=DESCENDING) WITH Period Age BMI_1 R□k_1
  /MODEL Period INTERCEPT=YES
 DISTRIBUTION=POISSON LINK=LOG
  /CRITERIA METHOD=FISHER(1) SCALE=1 COVB=ROBUST MAXITERATIONS=100 MAXSTEPHA
G=5
    PCONVERGE=1E-006(ABSOLUTE) SINGULAR=1E-012 ANALYSISTYPE=3(WALD) CILEVEL=95
YPE=WALD
    LIKELIHOOD=FULL
  /MISSING CLASSMISSING=EXCLUDE
  /PRINT CPS DESCRIPTIVES MODELINFO FIT SUMMARY SOLUTION (EXPONENTIATED).

* Generalized Linear Models.
GENLIN Apg5lth7 BY ParaKlass UtbClass (ORDER=DESCENDING) WITH Period Age BMI_1 R□k_1
  /MODEL Period ParaKlass Age BMI_1 R□k_1 UtbClass INTERCEPT=YES
 DISTRIBUTION=POISSON LINK=LOG
  /CRITERIA METHOD=FISHER(1) SCALE=1 COVB=ROBUST MAXITERATIONS=100 MAXSTEPHA
G=5
    PCONVERGE=1E-006(ABSOLUTE) SINGULAR=1E-012 ANALYSISTYPE=3(WALD) CILEVEL=95
YPE=WALD
    LIKELIHOOD=FULL
  /MISSING CLASSMISSING=EXCLUDE
  /PRINT CPS DESCRIPTIVES MODELINFO FIT SUMMARY SOLUTION (EXPONENTIATED).


* Generalized Linear Models.
GENLIN NICUmth3 BY ParaKlass (ORDER=DESCENDING) WITH Period Age BMI_1 R□k_1
```

```
 /MODEL Period INTERCEPT=YES
 DISTRIBUTION=POISSON LINK=LOG
 /CRITERIA METHOD=FISHER(1) SCALE=1 COVB=ROBUST MAXITERATIONS=100 MAXSTEPHA
G=5
   PCONVERGE=1E-006(ABSOLUTE) SINGULAR=1E-012 ANALYSISTYPE=3(WALD) CILEVEL=95
YPE=WALD
   LIKELIHOOD=FULL
 /MISSING CLASSMISSING=EXCLUDE
 /PRINT CPS DESCRIPTIVES MODELINFO FIT SUMMARY SOLUTION (EXPONENTIATED).


* Generalized Linear Models.
GENLIN NICUmth3 BY ParaKlass UtbClass (ORDER=DESCENDING) WITH Period Age BMI_1 R□k_
 /MODEL Period ParaKlass Age BMI_1 R□k_1 UtbClass INTERCEPT=YES
 DISTRIBUTION=POISSON LINK=LOG
 /CRITERIA METHOD=FISHER(1) SCALE=1 COVB=ROBUST MAXITERATIONS=100 MAXSTEPHA
G=5
   PCONVERGE=1E-006(ABSOLUTE) SINGULAR=1E-012 ANALYSISTYPE=3(WALD) CILEVEL=95
YPE=WALD
   LIKELIHOOD=FULL
 /MISSING CLASSMISSING=EXCLUDE
 /PRINT CPS DESCRIPTIVES MODELINFO FIT SUMMARY SOLUTION (EXPONENTIATED).


* Generalized Linear Models.
GENLIN MAS BY ParaKlass (ORDER=DESCENDING) WITH Period Age BMI_1 R□k_1
 /MODEL Period INTERCEPT=YES
 DISTRIBUTION=POISSON LINK=LOG
 /CRITERIA METHOD=FISHER(1) SCALE=1 COVB=ROBUST MAXITERATIONS=100 MAXSTEPHA
G=5
   PCONVERGE=1E-006(ABSOLUTE) SINGULAR=1E-012 ANALYSISTYPE=3(WALD) CILEVEL=95
YPE=WALD
   LIKELIHOOD=FULL
 /MISSING CLASSMISSING=EXCLUDE
 /PRINT CPS DESCRIPTIVES MODELINFO FIT SUMMARY SOLUTION (EXPONENTIATED).

* Generalized Linear Models.
GENLIN MAS BY ParaKlass UtbClass (ORDER=DESCENDING) WITH Period Age BMI_1 R□k_1
 /MODEL Period ParaKlass Age BMI_1 R□k_1 UtbClass INTERCEPT=YES
 DISTRIBUTION=POISSON LINK=LOG
 /CRITERIA METHOD=FISHER(1) SCALE=1 COVB=ROBUST MAXITERATIONS=100 MAXSTEPHA
G=5
   PCONVERGE=1E-006(ABSOLUTE) SINGULAR=1E-012 ANALYSISTYPE=3(WALD) CILEVEL=95
YPE=WALD
   LIKELIHOOD=FULL
 /MISSING CLASSMISSING=EXCLUDE
 /PRINT CPS DESCRIPTIVES MODELINFO FIT SUMMARY SOLUTION (EXPONENTIATED).


* Generalized Linear Models.
GENLIN Trauma BY ParaKlass (ORDER=DESCENDING) WITH Period Age BMI_1 R□k_1
```

```
  /MODEL Period INTERCEPT=YES
 DISTRIBUTION=POISSON LINK=LOG
  /CRITERIA METHOD=FISHER(1) SCALE=1 COVB=ROBUST MAXITERATIONS=100 MAXSTEPHA
G=5
    PCONVERGE=1E-006(ABSOLUTE) SINGULAR=1E-012 ANALYSISTYPE=3(WALD) CILEVEL=95
YPE=WALD
    LIKELIHOOD=FULL
  /MISSING CLASSMISSING=EXCLUDE
  /PRINT CPS DESCRIPTIVES MODELINFO FIT SUMMARY SOLUTION (EXPONENTIATED).


* Generalized Linear Models.
GENLIN Trauma BY ParaKlass UtbClass (ORDER=DESCENDING) WITH Period Age BMI_1 R□k_1
  /MODEL Period ParaKlass Age BMI_1 R□k_1 UtbClass INTERCEPT=YES
 DISTRIBUTION=POISSON LINK=LOG
  /CRITERIA METHOD=FISHER(1) SCALE=1 COVB=ROBUST MAXITERATIONS=100 MAXSTEPHA
G=5
    PCONVERGE=1E-006(ABSOLUTE) SINGULAR=1E-012 ANALYSISTYPE=3(WALD) CILEVEL=95
YPE=WALD
    LIKELIHOOD=FULL
  /MISSING CLASSMISSING=EXCLUDE
  /PRINT CPS DESCRIPTIVES MODELINFO FIT SUMMARY SOLUTION (EXPONENTIATED).

* Generalized Linear Models.
GENLIN AnyHIE BY ParaKlass (ORDER=DESCENDING) WITH Period Age BMI_1 R□k_1
  /MODEL Period INTERCEPT=YES
 DISTRIBUTION=POISSON LINK=LOG
  /CRITERIA METHOD=FISHER(1) SCALE=1 COVB=ROBUST MAXITERATIONS=100 MAXSTEPHA
G=5
    PCONVERGE=1E-006(ABSOLUTE) SINGULAR=1E-012 ANALYSISTYPE=3(WALD) CILEVEL=95
YPE=WALD
    LIKELIHOOD=FULL
  /MISSING CLASSMISSING=EXCLUDE
  /PRINT CPS DESCRIPTIVES MODELINFO FIT SUMMARY SOLUTION (EXPONENTIATED).

* Generalized Linear Models.
GENLIN AnyHIE BY ParaKlass UtbClass (ORDER=DESCENDING) WITH Period Age BMI_1 R□k_1
  /MODEL Period ParaKlass Age BMI_1 R□k_1 UtbClass INTERCEPT=YES
 DISTRIBUTION=POISSON LINK=LOG
  /CRITERIA METHOD=FISHER(1) SCALE=1 COVB=ROBUST MAXITERATIONS=100 MAXSTEPHA
G=5
    PCONVERGE=1E-006(ABSOLUTE) SINGULAR=1E-012 ANALYSISTYPE=3(WALD) CILEVEL=95
YPE=WALD
    LIKELIHOOD=FULL
  /MISSING CLASSMISSING=EXCLUDE
  /PRINT CPS DESCRIPTIVES MODELINFO FIT SUMMARY SOLUTION (EXPONENTIATED).

* Generalized Linear Models.
GENLIN HIE2_3 BY ParaKlass (ORDER=DESCENDING) WITH Period Age BMI_1 R□k_1
  /MODEL Period INTERCEPT=YES
 DISTRIBUTION=POISSON LINK=LOG
```

```
  /CRITERIA METHOD=FISHER(1) SCALE=1 COVB=ROBUST MAXITERATIONS=100 MAXSTEPHA
G=5
    PCONVERGE=1E-006(ABSOLUTE) SINGULAR=1E-012 ANALYSISTYPE=3(WALD) CILEVEL=95
YPE=WALD
    LIKELIHOOD=FULL
  /MISSING CLASSMISSING=EXCLUDE
  /PRINT CPS DESCRIPTIVES MODELINFO FIT SUMMARY SOLUTION (EXPONENTIATED).

* Generalized Linear Models.
GENLIN HIE2_3 BY ParaKlass UtbClass (ORDER=DESCENDING) WITH Period Age BMI_1 R□k_1
  /MODEL Period ParaKlass Age BMI_1 R□k_1 UtbClass INTERCEPT=YES
 DISTRIBUTION=POISSON LINK=LOG
  /CRITERIA METHOD=FISHER(1) SCALE=1 COVB=ROBUST MAXITERATIONS=100 MAXSTEPHA
G=5
    PCONVERGE=1E-006(ABSOLUTE) SINGULAR=1E-012 ANALYSISTYPE=3(WALD) CILEVEL=95
YPE=WALD
    LIKELIHOOD=FULL
  /MISSING CLASSMISSING=EXCLUDE
  /PRINT CPS DESCRIPTIVES MODELINFO FIT SUMMARY SOLUTION (EXPONENTIATED).


*==========================================================================
=====
* Maternal outcomes, secondary outcomes

USE ALL.

* Generalized Linear Models.
GENLIN Instrumental BY ParaKlass (ORDER=DESCENDING) WITH Period Age BMI_1 R□k_1
  /MODEL Period  INTERCEPT=YES
 DISTRIBUTION=POISSON LINK=LOG
  /CRITERIA METHOD=FISHER(1) SCALE=1 COVB=ROBUST MAXITERATIONS=100 MAXSTEPHA
G=5
    PCONVERGE=1E-006(ABSOLUTE) SINGULAR=1E-012 ANALYSISTYPE=3(WALD) CILEVEL=95
YPE=WALD
    LIKELIHOOD=FULL
  /MISSING CLASSMISSING=EXCLUDE
  /PRINT CPS DESCRIPTIVES MODELINFO FIT SUMMARY SOLUTION (EXPONENTIATED).

* Generalized Linear Models.
GENLIN Instrumental BY ParaKlass UtbClass (ORDER=DESCENDING) WITH Period Age BMI_1
R□k_1
  /MODEL Period ParaKlass Age BMI_1 R□k_1 UtbClass INTERCEPT=YES
 DISTRIBUTION=POISSON LINK=LOG
  /CRITERIA METHOD=FISHER(1) SCALE=1 COVB=ROBUST MAXITERATIONS=100 MAXSTEPHA
G=5
    PCONVERGE=1E-006(ABSOLUTE) SINGULAR=1E-012 ANALYSISTYPE=3(WALD) CILEVEL=95
YPE=WALD
    LIKELIHOOD=FULL
  /MISSING CLASSMISSING=EXCLUDE
  /PRINT CPS DESCRIPTIVES MODELINFO FIT SUMMARY SOLUTION (EXPONENTIATED).
```

```
* Generalized Linear Models.
GENLIN VagNonInstr BY ParaKlass (ORDER=DESCENDING) WITH Period Age BMI_1 R□k_1
  /MODEL Period  INTERCEPT=YES
 DISTRIBUTION=POISSON LINK=LOG
  /CRITERIA METHOD=FISHER(1) SCALE=1 COVB=ROBUST MAXITERATIONS=100 MAXSTEPHA
G=5
    PCONVERGE=1E-006(ABSOLUTE) SINGULAR=1E-012 ANALYSISTYPE=3(WALD) CILEVEL=95
YPE=WALD
    LIKELIHOOD=FULL
  /MISSING CLASSMISSING=EXCLUDE
  /PRINT CPS DESCRIPTIVES MODELINFO FIT SUMMARY SOLUTION (EXPONENTIATED).

* Generalized Linear Models.
GENLIN VagNonInstr BY ParaKlass UtbClass (ORDER=DESCENDING) WITH Period Age BMI_1
R□k_1
  /MODEL Period ParaKlass Age BMI_1 R□k_1 UtbClass INTERCEPT=YES
 DISTRIBUTION=POISSON LINK=LOG
  /CRITERIA METHOD=FISHER(1) SCALE=1 COVB=ROBUST MAXITERATIONS=100 MAXSTEPHA
G=5
    PCONVERGE=1E-006(ABSOLUTE) SINGULAR=1E-012 ANALYSISTYPE=3(WALD) CILEVEL=95
YPE=WALD
    LIKELIHOOD=FULL
  /MISSING CLASSMISSING=EXCLUDE
  /PRINT CPS DESCRIPTIVES MODELINFO FIT SUMMARY SOLUTION (EXPONENTIATED).


USE ALL.
COMPUTE filter_$=(FS=1 or FS>3).
VARIABLE LABELS filter_$ 'FS=1 or FS>3 (FILTER)'.
VALUE LABELS filter_$ 0 'Not Selected' 1 'Selected'.
FORMATS filter_$ (f1.0).
FILTER BY filter_$.
EXECUTE.

* Generalized Linear Models.
GENLIN Sfinkter BY ParaKlass (ORDER=DESCENDING) WITH Period Age BMI_1 R□k_1
  /MODEL Period  INTERCEPT=YES
 DISTRIBUTION=POISSON LINK=LOG
  /CRITERIA METHOD=FISHER(1) SCALE=1 COVB=ROBUST MAXITERATIONS=100 MAXSTEPHA
G=5
    PCONVERGE=1E-006(ABSOLUTE) SINGULAR=1E-012 ANALYSISTYPE=3(WALD) CILEVEL=95
YPE=WALD
    LIKELIHOOD=FULL
  /MISSING CLASSMISSING=EXCLUDE
  /PRINT CPS DESCRIPTIVES MODELINFO FIT SUMMARY SOLUTION (EXPONENTIATED).

* Generalized Linear Models.
GENLIN Sfinkter BY ParaKlass UtbClass (ORDER=DESCENDING) WITH Period Age BMI_1 R□k_1
  /MODEL Period ParaKlass Age BMI_1 R□k_1 UtbClass INTERCEPT=YES
 DISTRIBUTION=POISSON LINK=LOG
```

```
  /CRITERIA METHOD=FISHER(1) SCALE=1 COVB=ROBUST MAXITERATIONS=100 MAXSTEPHA
G=5
    PCONVERGE=1E-006(ABSOLUTE) SINGULAR=1E-012 ANALYSISTYPE=3(WALD) CILEVEL=95
YPE=WALD
    LIKELIHOOD=FULL
  /MISSING CLASSMISSING=EXCLUDE
  /PRINT CPS DESCRIPTIVES MODELINFO FIT SUMMARY SOLUTION (EXPONENTIATED).


USE ALL.

* Generalized Linear Models.
GENLIN Endometrit BY ParaKlass UtbClass (ORDER=DESCENDING) WITH Period Age BMI_1 R□k_
  /MODEL Period  INTERCEPT=YES
 DISTRIBUTION=POISSON LINK=LOG
  /CRITERIA METHOD=FISHER(1) SCALE=1 COVB=ROBUST MAXITERATIONS=100 MAXSTEPHA
G=5
    PCONVERGE=1E-006(ABSOLUTE) SINGULAR=1E-012 ANALYSISTYPE=3(WALD) CILEVEL=95
YPE=WALD
    LIKELIHOOD=FULL
  /MISSING CLASSMISSING=EXCLUDE
  /PRINT CPS DESCRIPTIVES MODELINFO FIT SUMMARY SOLUTION (EXPONENTIATED).

* Generalized Linear Models.
GENLIN Endometrit BY ParaKlass UtbClass (ORDER=DESCENDING) WITH Period Age BMI_1 R□k_
  /MODEL Period ParaKlass Age BMI_1 R□k_1 UtbClass INTERCEPT=YES
 DISTRIBUTION=POISSON LINK=LOG
  /CRITERIA METHOD=FISHER(1) SCALE=1 COVB=ROBUST MAXITERATIONS=100 MAXSTEPHA
G=5
    PCONVERGE=1E-006(ABSOLUTE) SINGULAR=1E-012 ANALYSISTYPE=3(WALD) CILEVEL=95
YPE=WALD
    LIKELIHOOD=FULL
  /MISSING CLASSMISSING=EXCLUDE
  /PRINT CPS DESCRIPTIVES MODELINFO FIT SUMMARY SOLUTION (EXPONENTIATED).


* Generalized Linear Models.
GENLIN Blod1000 BY ParaKlass UtbClass (ORDER=DESCENDING) WITH Period Age BMI_1 R□k_1
  /MODEL Period  INTERCEPT=YES
 DISTRIBUTION=POISSON LINK=LOG
  /CRITERIA METHOD=FISHER(1) SCALE=1 COVB=ROBUST MAXITERATIONS=100 MAXSTEPHA
G=5
    PCONVERGE=1E-006(ABSOLUTE) SINGULAR=1E-012 ANALYSISTYPE=3(WALD) CILEVEL=95
YPE=WALD
    LIKELIHOOD=FULL
  /MISSING CLASSMISSING=EXCLUDE
  /PRINT CPS DESCRIPTIVES MODELINFO FIT SUMMARY SOLUTION (EXPONENTIATED).

* Generalized Linear Models.
GENLIN Blod1000 BY ParaKlass UtbClass (ORDER=DESCENDING) WITH Period Age BMI_1 R□k_1
  /MODEL Period ParaKlass Age BMI_1 R□k_1 UtbClass INTERCEPT=YES
```

```
 DISTRIBUTION=POISSON LINK=LOG
  /CRITERIA METHOD=FISHER(1) SCALE=1 COVB=ROBUST MAXITERATIONS=100 MAXSTEPHA
G=5
    PCONVERGE=1E-006(ABSOLUTE) SINGULAR=1E-012 ANALYSISTYPE=3(WALD) CILEVEL=95
YPE=WALD
    LIKELIHOOD=FULL
  /MISSING CLASSMISSING=EXCLUDE
  /PRINT CPS DESCRIPTIVES MODELINFO FIT SUMMARY SOLUTION (EXPONENTIATED).

*
=============================================================================
===
*20240404 Patient satisfaction.

* Generalized Linear Models.
GENLIN PoorVASforl BY ParaKlass UtbClass (ORDER=DESCENDING) WITH Period Age BMI_1
R□k_1
  /MODEL Period INTERCEPT=YES
 DISTRIBUTION=POISSON LINK=LOG
  /CRITERIA METHOD=FISHER(1) SCALE=1 COVB=ROBUST MAXITERATIONS=100 MAXSTEPHA
G=5
    PCONVERGE=1E-006(ABSOLUTE) SINGULAR=1E-012 ANALYSISTYPE=3(WALD) CILEVEL=95
YPE=WALD
    LIKELIHOOD=FULL
  /MISSING CLASSMISSING=EXCLUDE
  /PRINT CPS DESCRIPTIVES MODELINFO FIT SUMMARY SOLUTION (EXPONENTIATED).


* Generalized Linear Models.
GENLIN PoorVASforl BY ParaKlass UtbClass (ORDER=DESCENDING) WITH Period Age BMI_1
R□k_1
  /MODEL Period ParaKlass Age BMI_1 R□k_1 UtbClass INTERCEPT=YES
 DISTRIBUTION=POISSON LINK=LOG
  /CRITERIA METHOD=FISHER(1) SCALE=1 COVB=ROBUST MAXITERATIONS=100 MAXSTEPHA
G=5
    PCONVERGE=1E-006(ABSOLUTE) SINGULAR=1E-012 ANALYSISTYPE=3(WALD) CILEVEL=95
YPE=WALD
    LIKELIHOOD=FULL
  /MISSING CLASSMISSING=EXCLUDE
  /PRINT CPS DESCRIPTIVES MODELINFO FIT SUMMARY SOLUTION (EXPONENTIATED).

* Generalized Linear Models.
GENLIN PoorSelfHealthpp BY ParaKlass UtbClass (ORDER=DESCENDING) WITH Period Age BMI_
R□k_1
  /MODEL Period  INTERCEPT=YES
 DISTRIBUTION=POISSON LINK=LOG
  /CRITERIA METHOD=FISHER(1) SCALE=1 COVB=ROBUST MAXITERATIONS=100 MAXSTEPHA
G=5
    PCONVERGE=1E-006(ABSOLUTE) SINGULAR=1E-012 ANALYSISTYPE=3(WALD) CILEVEL=95
YPE=WALD
    LIKELIHOOD=FULL
```

```
  /MISSING CLASSMISSING=EXCLUDE
  /PRINT CPS DESCRIPTIVES MODELINFO FIT SUMMARY SOLUTION (EXPONENTIATED).


* Generalized Linear Models.
GENLIN PoorSelfHealthpp BY ParaKlass UtbClass (ORDER=DESCENDING) WITH Period Age BMI_1
R□k_1
  /MODEL Period ParaKlass Age BMI_1 R□k_1 UtbClass INTERCEPT=YES
 DISTRIBUTION=POISSON LINK=LOG
  /CRITERIA METHOD=FISHER(1) SCALE=1 COVB=ROBUST MAXITERATIONS=100 MAXSTEPHA
G=5
    PCONVERGE=1E-006(ABSOLUTE) SINGULAR=1E-012 ANALYSISTYPE=3(WALD) CILEVEL=95
YPE=WALD
    LIKELIHOOD=FULL
  /MISSING CLASSMISSING=EXCLUDE
  /PRINT CPS DESCRIPTIVES MODELINFO FIT SUMMARY SOLUTION (EXPONENTIATED).
```
