# Supplementary material for: Maternal and perinatal outcomes after implementation of a more active management in late- and postterm pregnancies in Sweden: A population-based cohort study
Source: PLoS Med. 2025 Jan 16;22(1):e1004504. doi: 10.1371/journal.pmed.1004504 (PMC11737695; doi:10.1371/journal.pmed.1004504)
Supplement: S1 Table — (DOCX) [file pmed.1004504.s004.docx]

S1 Table. Results from analyses based on weighted summary of unit-specific Risk Ratios (RR) (period 2 versus period 1). Significant findings are indicated in bold text.

|  |  | Crude | | Adjusted | |
| --- | --- | --- | --- | --- | --- |
|  |  | RR | 95% CI | RR | 95% CI |
| Primary outcomes | |  |  |  |  |
|  | Peri/Neonatal death | 0.60 | 0.44; 0.82 | **0.60** | **0.44; 0.82** |
|  | Composite outcome* | 0.94 | 0.90; 0.98 | **0.92** | **0.88; 0.96** |
|  | Composite II^†^ | 0.83 | 0.77; 0.89 | **0.80** | **0.75; 0.86** |
|  |  |  |  |  |  |
|  | Emergency caesarean section | 1.12 | 1.09; 1.16 | 1.07 | 1.04; 1.10 |

CI=confidence interval, HIE= hypoxic ischaemic encephalopathy, NICU= neonatal intensive care unit

^*^Composite outcome: Peri/neonatal death, Apgar 5 minute 0-3, NICU ≥4 days, HIE 1-3, Meconium aspiration, or delivery trauma.

^†^Composite II: Like Composite, but without criteria NICU ≥4 days
