# Supplementary material for: Maternal and perinatal outcomes after implementation of a more active management in late- and postterm pregnancies in Sweden: A population-based cohort study
Source: PLoS Med. 2025 Jan 16;22(1):e1004504. doi: 10.1371/journal.pmed.1004504 (PMC11737695; doi:10.1371/journal.pmed.1004504)
Supplement: S2 Table — (DOCX) [file pmed.1004504.s005.docx]

S 2 Table. Risk for peri/neonatal deaths by study period. Sensitivity analyses excluding births during 2017 and infants with congenital malformations, respectively. Significant findings are indicated in bold text.

|  |  | Delivery period | | | |  | Risk Ratio (RR) period 2 versus period 1 | | | |
| --- | --- | --- | --- | --- | --- | --- | --- | --- | --- | --- |
|  |  | 1 | | 2 | |  | Crude | | Adjusted^†^ | |
|  |  | n | Per thousand | n | Per thousand |  | RR | 95% CI; p-value | RR | 95% CI; p-value |
| **Excluding births 2017 (n=23 946)** | | 2018-2019  N=47 619 | | 2020-2023  N=78 805 | |  |  |  |  |  |
|  | Peri/neonatal deaths | 83 | 1.7 | 74 | 0.9 |  | **0.54** | **0.40, 0.75; p<0.001** | **0.53** | **0.39, 0.72; p<0.001** |
|  |  |  |  |  |  |  |  |  |  |  |
| **Excluding malformations (n=3 113)** | | 2017-2019  N=69 985 | | 2020-2023  N=77 272 | |  |  |  |  |  |
|  | Peri/neonatal deaths | 115 | 1.6 | 69 | 0.9 |  | **0.56** | **0.41, 0.75; p<0.001** | **0.54** | **0.40, 0.74; p<0.001** |
