# Supplementary material for: Maternal and perinatal outcomes after implementation of a more active management in late- and postterm pregnancies in Sweden: A population-based cohort study
Source: PLoS Med. 2025 Jan 16;22(1):e1004504. doi: 10.1371/journal.pmed.1004504 (PMC11737695; doi:10.1371/journal.pmed.1004504)
Supplement: Attachment — Submitted filename: kallen.pdf [file pmed.1004504.s006.pdf]

This paper uses registry data to examine a change in policy about management of late–term pregnancies in Sweden.

I was asked for a methodological report and I interpret that to include all aspects of the design and conduct of the study.

Points of detail

Page 11 Imputing a single value is not the best strategy although without knowing more about the distribution and amount missing it is hard to be sure. From Table 1 depending on how the missing is distributed between BMI and smoking there might be enough to justify multiple imputation.

Page 12 I think there is a typo here as I assume it was $\log(RR)$ which was modelled.

Page 12 Although quartal seems to exist it is much more usual to use quarter in English. In fact I have never seen quartal in writing nor heard it in any of the dialects of regions in England where I have lived.

Page 13 Patient involvement in interpretation might have been helpful to distinguish between outcome differences which were merely statistically significant and those which were also clinically important. Having said that for some of the outcomes in Table 2 like stillbirth and peri– or neo–natal death it is hard to imaging anyone thinking a halving of risk is unimportant even for a mercifully rare outcome.

Page 14 Presumably the failure to find differences at 42^{+0} is because there were relatively few deliveries? That is what the figure suggests to me. If true perhaps point it out here.

Page 20 Perhaps it would be instructive to give the risk reduction from the trials here and repeat the present study finding so that readers can make an immediate comparison.

Page 23 Why exclude the hospitals with low response rate? It seems rather unfair that your data is thrown away if other women attending your hospital have failed to respond. If I found that my responses had been dropped in similar circumstances I would have been disappointed.

Page 28 It might be helpful, even in this age of machine translation, to give titles in English as well as Swedish. This has been done for reference 11 but not for 23, 24 and 29.

Page 31, Table 1 As is pointed out on page 15 these differences are relatively small but because of the substantial sample size they all achieve statistical significance. I would prefer some measure of effect with confidence interval to illustrate this but for $r \times c$ tables these are unsatisfactory. I do not think the p–values add anything here. I understand that editorial policy is to always have p–values but the radical step here is to ask for a waiver of that requirement, delete them, and let p–addicts calculate them themselves.

Supplement I am afraid this has left me completely baffled. I do not understand what the table entries are and it is not helped by splitting two–digit numbers across rows. I also find the colouring hard to understand.

Point of more substance

If, as page 8 suggests, there might be differences in hospital practice I wonder whether the present analyses which ignore hospital are optimal. I think I would have included a random intercept for hospital and considered whether there were other hospital–level covariates which were worth investigating.

Summary

Mostly for clarification and enhancement.

Michael Dewey
