# Supplementary material for: Maternal and perinatal outcomes after implementation of a more active management in late- and postterm pregnancies in Sweden: A population-based cohort study
Source: PLoS Med. 2025 Jan 16;22(1):e1004504. doi: 10.1371/journal.pmed.1004504 (PMC11737695; doi:10.1371/journal.pmed.1004504)
Supplement: Attachment — Submitted filename: Answers Referee PLoS M D-24-01706 v 41.docx [file pmed.1004504.s007.docx]

Dear Editor,

Dr Philippa Dodd,

We thank you and the reviewers for your valuable comments and the opportunity to improve and resubmit our work.

In this re-submission, we provide you with a point-by-point response to the reviewer’s comments (in italics), and the manuscript in a “Tracked changes” format as well as a clean version. The page and line numbers in our quotes below refer to the version with tracked changes.

All authors have read and approved the re-submission of the manuscript; the manuscript has not been published and is not being considered for publication elsewhere, in whole or in part, in any language.

**Comments from the reviewers:
Reviewer #1:**

Kallén et al June 13, 2024. This paper uses registry data to examine a change in policy about management of late–term pregnancies in Sweden. I was asked for a methodological report and I interpret that to include all aspects of the design and conduct of the study.

***Answer:*** *We thank this reviewer for his thorough report, and we have done our best to adjust the analyses, tables, and text according to the advice.*

Points of detail Page 11

Imputing a single value is not the best strategy although without knowing more about the distribution and amount missing it is hard to be sure. From Table 1 depending on how the missing is distributed between BMI and smoking there might be enough to justify multiple imputation.

***Answer:*** *We performed mean-value imputation since we expected the impact of the possible confounders on the results to be small, and the missing number rates were low. However, following the referee’s advice, we re-analysed the main analyses using multiple imputation, and these results are now displayed in the Tables and in the Result section. As the difference between the new and the original RRs never exceeded 0.01, we kept the results retrieved after mean imputation for the sensitivity analyses.*

*In the Method section page 12, line 230 we have added: “****In the main analyses (primary outcomes), multiple imputation was used to replace missing information on BMI or smoking. In the other analyses,*** *missing information on BMI and smoking was replaced by the overall mean.”*

*We have also pointed out this in the Method section as an additional analysis not included in the original study protocol but suggested by peer review comments, page 12, line 217****. “Changes from the planned protocol as response to peer review comments were 1. multiple imputation used to replace missing information on BMI or smoking for the main outcomes and 2. the additional analysis to control for possible difference in hospital practice.”***

Page 12 I think there is a typo here as I assume it was log(RR) which was modelled.

***Answer:*** *Yes, thank you. We forgot to specify log(RR) in one of the sentences. Corrected now. Page 12, line 234*

Page 12 Although quartal seems to exist it is much more usual to use quarter in English. In fact I have never seen quartal in writing nor heard it in any of the dialects of regions in England where I have lived.

***Answer:*** *We have changed “quartal” to “quarter” in the whole manuscript.*

Page 13 Patient involvement in interpretation might have been helpful to distinguish between outcome differences which were merely statistically significant and those which were also clinically important. Having said that for some of the outcomes in Table 2 like stillbirth and peri– or neo–natal death it is hard to imaging anyone thinking a halving of risk is unimportant even for a mercifully rare outcome.

***Answer:*** *We agree that patient involvement in clinical studies is always important and for main outcome we also think that a 50% reduction in mortality would be important for any patient.*

Page 14 Presumably the failure to find differences at 42+0 is because there were relatively few deliveries? That is what the figure suggests to me. If true perhaps point it out here.

***Answer:*** *We assume that the reviewer refers to Figure 2 and we have added more data:*

*Page 15, line 292: “Figure 2 (left) shows the induction rate by time period and gestational week (three-days interval) on the x-axis. A very prominent increase of the induction rate between the two time periods (2017 – 2019 and 2020 – 2023, respectively) was noted among births at 41^+1^ to 41^+6^ weeks* ***(from 21% to 55%, p<0.001)****. Among deliveries at 39^+0^ to 41^+0^ weeks, the induction rate was significantly higher during period 2 than period 1* ***(from 14% to 20%, p<0.001),*** *but among deliveries at 42^+0^ weeks or more, no* ***major*** *change in induction rates between the two study periods was observed* ***(from 77% to 82%, p<0.001).***

Page 20 Perhaps it would be instructive to give the risk reduction from the trials here and repeat the present study finding so that readers can make an immediate comparison.

***Answer:*** *We have added adjusted RR and 95% CI for perinatal mortality for our study and for the referred systematic review with individual participant data meta-analysis by Alkmark et al according to the instructions from the referee.*

*Page 22, line 446: “Our finding of reduced perinatal mortality (****adjusted RR 0.52; 95% CI 0.38-0.69)*** *after changing to an active management of late- and postterm pregnancies are in line with results from a systematic review which has evaluated induction at 41 weeks vs expectant management and induction at 42 weeks in three randomised trials (N=5161 women). Data from the two largest trials (N=4561 women) were included in an individual participant data meta-analysis in this review. ^12^ Early induction significantly reduced the composite outcome of perinatal mortality and severe neonatal morbidity and perinatal mortality alone* ***(perinatal mortality: OR 0.21; 95% CI 0.06-0.78)*** *without increasing the caesarean section rate or maternal morbidity”.*

Page 23 Why exclude the hospitals with low response rate? It seems rather unfair that your data is thrown away if other women attending your hospital have failed to respond. If I found that my responses had been dropped in similar circumstances I would have been disappointed.

**Answer:** *We performed the analyses according to the recommendations from the Swedish Pregnancy Register (SPR). (ref 29 Petersson K SY, Conner P, Sengpiel V, et al. Graviditetsregistrets årsrapport 2021 2022). However, we have followed the advice from this reviewer, and in the revised version, we have included all valid results in the analyses and deleted reference 29. Table 3 and Results, page 18, line 359*

*“In this cohort of women giving birth at 41^+0^ weeks or more, the proportion of women who reported a negative childbirth experience was slightly lower among women giving birth in period 2 compared to period 1* ***(6.5% as compared to 7.5% in the first period, adjusted RR 0.87; 95% CI 0.83-0.91, p<0.001).*** *The proportion of women who reported a poor self-rated health after childbirth* ***(7.9% vs 7.5%)*** *did not change between the periods* ***(adjusted RR1.02; 95% CI 0.98-1.06, p=0.274).***

Page 28 It might be helpful, even in this age of machine translation, to give titles in English as well as Swedish. This has been done for reference 11 but not for 23, 24 and 29.

***Answer:*** *Reference 29 has been deleted. Unfortunately, titles for references 23 and 24 are not available in English since they are national guidelines written in Norwegian and Swedish, respectively.*

Page 1 Kallén et al June 13, 2024 Page 31, Table 1 As is pointed out on page 15 these differences are relatively small but because of the substantial sample size they all achieve statistical significance. I would prefer some measure of effect with confidence interval to illustrate this but for r × c tables these are unsatisfactory. I do not think the p–values add anything here. I understand that editorial policy is to always have p–values but the radical step here is to ask for a waiver of that requirement, delete them, and let p–addicts calculate them themselves.

***Answer:*** *We agree with the referee that the p-values are of little interest as even small differences between the periods become statistically significant. The p-values are removed from table 1, if the editor agrees. Moreover, we have deleted the following text accordingly in the Result section, page 16, line 315: “~~Compared to women giving birth during the first period, women giving birth during the later period were slightly older, more often primiparous, more often had an early-pregnancy BMI of 30 or more, gestational diabetes, gestational hypertension or preeclampsia, and had more often conceived after assisted reproduction, but were less often smoking in early pregnancy, less often gave birth to infants of male sex, and less often gave birth to infants weighing 4500g or more.~~ ~~Almost all changes of characteristics reached statistical significance (data not shown),~~ but ~~the differences between the birth year cohort~~* *~~characteristics were small in absolute measures.~~* And the text is now as follows, page 15, line 286***:***

***“Table 1 shows the maternal and pregnancy characteristics by period of delivery (2017 – 2019 or 2020 – 2023) among pregnancies lasting 41^+0^ weeks or more. The differences in characteristics by period of delivery were small in absolute measures.”***

Supplement I am afraid this has left me completely baffled. I do not understand what the table entries are and it is not helped by splitting two–digit numbers across rows. I also find the colouring hard to understand.

***Answer:*** *We provided this table to illustrate the magnitude and the nature of the policy-change among the hospitals, and also to explain the hospital-specific breaking points. The S1Table is now removed, if the editor agrees.*

Point of more substance If, as page 8 suggests, there might be differences in hospital practice I wonder whether the present analyses which ignore hospital are optimal.

I think I would have included a random intercept for hospital and considered whether there were other hospital–level covariates which were worth investigating.

***Answer:*** *“We understand this comment, and our intention was to perform GEE -analyses to control for variation within and between hospitals. However, the GEE models did not perform very well when applied to this large data set. Instead, in the revised version, we performed additional analyses in which we controlled for possible difference in hospital practice, by stratifying the results by the k units, and compute an overall RR by weighing the unit-specific log (RRs) by precision (1/Standard Error of log (RR_k_)). The results from these analyses were close to the original estimates, and therefore we assume that the policy difference between units had no major impact on the results regarding difference between period 2 vs period 1. The results are displayed in a supplementary table (S1 table) and are also mentioned in the Results section, page 17, line 340, and the analysis is presented in the Methods section page 12, line 222:*

***“To control for possible difference in hospital practice, additional analyses were carried out where the results before and after the breaking-point were stratified by the k units, and overall RRs were computed in which the unit-specific log(RRs) were weighted by precision (1/Standard Error of log (RR_k_)).”***

*We have also pointed out this in the Method section as an additional analysis not included in the original study protocol but suggested by peer review comments, page 12, line 218:*

***“Changes from the planned protocol as response to peer review comments were 1. multiple imputation used to replace missing information on BMI or smoking for the main outcomes and 2. the additional analysis to control for possible difference in hospital practice.”***

Reviewer #2:

Reviewer #2: Thank you for sending me this paper to review.  The authors are to be congratulated on their commitment to evaluating what often goes unevaluated, policy change, and for including women's experiences in their evaluation.
Overall however I am not sure that this manuscript adds substantially to the existing evidence regarding induction of labour. The authors report a change in practice between two time periods and then a change in outcomes.  These time periods also encompass the pandemic and insufficient reference is made to this.  The methods do not account sufficiently for comorbidity and the results are not clearly stated.  The authors do not make sufficient reference to the background literature and context - they state that the observed increase in induction at 39 weeks needs further investigation, but do not reference the 35/39 trial (published 2016) and ARRIVE trial (published 2018) which both made the offer of induction at 39 weeks much more common across the world. I think it is a well conducted study and with revision is suitable for publication in another journal, but at present, I do not think it meets the high standards of PLOS Medicine.

***Answer:*** *Thank you for the positive comment but also* *for pointing out the need for further clarification and discussion! We believe that the value of real-world follow-up studies should not be underestimated and are complementary to previously published RCTs and IPD-MA reports and as commented by reviewer 3: “population-based real-word data research contributes significantly to decisions in clinical practice.” The present study has some methodological shortcomings as commented in the discussion but also important advantages as it is population-based, almost complete follow-up of cases and includes sufficiently large cohorts in relation to the rare but clinically important outcomes such as perinatal mortality.*

*Concerning the more specific comments we have added some more data on the pandemic, comorbidity, and revised the presentation of results, and we have also commented on the two large trials, 35/39 (2016) and ARRIVE (2018):*

***Answer:***

1. *Concerning the pandemic we have added additional references about the possible influence of the pandemic on the results (To el al.; 2024,* *doi:10.1371/journal.pone.0303175, Silva et al., 2023, doi: 10.1055/s-0043-1772182. Kurbatfinksi et al., 2024, doi: 10.1016/j.heliyon.2024.e34165. Page 22, line 438:*

***“A higher rate of caesarean section during the COVID-19 pandemic period 2020-2022 than before the pandemic has been reported in some studies (Gurol-Urganci et al., To et al., doi: 10.1371/journal.pone.0303175, doi: 10.1371/journal.pmed.100388, Silva et al., doi: 10.1055/s-0043-1772182), although other studies have not found any change (Chmielewska et al., doi: 10.1016/s2214-109x(21)00079-6, Kurbatfinski et al, doi: 10.1016/j.heliyon.2024.e34165). “***

*2. The methods do not account sufficiently for comorbidity: Please see Answer below, Statistical methods*

*3. “…and the results are not clearly stated.” Please see Answer below concerning revision of the result section where we have added RRs, CIs, and p-values.*

*4. More background literature has been added in introduction and discussion, and the ARRIVE trial and the 35/39 trial are now cited. The reason for not mentioning them in our first submission was that we focused on trials on induction at gestational week 41 and later.*

Introduction:

*Page 7, line 111:“Induction of labour (induction) is used to reduce these adverse effects* ***as shown in the ARRIVE trial (Grobman et al, doi: 10.1056/NEJMoa1800566)*** *but is controversial since* ***in observational studies*** *the procedure, when compared to spontaneous onset of labour, has been associated with harm such as increased need for interventions during labour, uterine rupture, and adverse neonatal outcome.*

*Page 7, line 119: Systematic reviews and randomised clinical trials suggest that induction before 42 gestational weeks may reduce perinatal mortality and severe neonatal morbidity without increasing the frequency of caesarean section, operative vaginal delivery, or postpartum haemorrhage. ^10-15^* ***Furthermore, the ARRIVE trial showed that induction at 39 weeks in low-risk nulliparous women resulted in a significantly lower frequency of caesarean section but did not change composite adverse perinatal outcome (Grobman et al., doi: 10.1056/NEJMoa1800566).***

Discussion:

*Page 27, line 554:* ***However, results from the 35/39 trial in the UK (Walker et al, doi: 10.1056/NEJMoa1509117) and the ARRIVE trial in the USA (Grobman et al., doi: 10.1056/NEJMoa1800566) published in 2016 and 2018, respectively, which both offered elective induction in nulliparous women at 39 weeks of gestation compared with expectant management, may also have had implications for obstetric clinical practice in Sweden and other countries across the world.”***

Specific comments:
Abstract: should state what the policy was prior to the change in the background section, at present this could only be inferred from the results.

***Answer****: We have added the policy prior to the change in the background section*

*of the abstract.*

*Page 4, line 58: “We evaluated maternal and perinatal outcomes after a national shift in Sweden* ***from expectancy and induction at 42+0 weeks*** *to a more active management offering induction from 41+0 weeks or an individual plan aiming at birth or active labour no later than 42+0 weeks.”*

Introduction:
Lines 104-107: the existing evidence does not suggest a dichotomy (as suggested here) but rather, a risk that increases with advancing gestational age.

***Answer:*** *We have revised the text.*

*Page 7, line 110: “Rare but severe outcomes such as stillbirth, perinatal mortality, and severe neonatal and maternal morbidity are more common among prolonged pregnancies than among full term pregnancies (between 39 weeks+0 days (39^+0^ weeks) and 40 weeks+6 days (40^+6^ weeks)* ***and increases as gestational age advances.”***

Lines 108-111: The literature does not universally confirm this to be the case, for example, the ARRIVE trial (doi 10.1056/NEJMoa1800566).

***Answer:*** *As mentioned above we have revised the text in the Introduction:*

*Page 7, line 111: “Induction of labour (induction) is used to reduce these adverse effects* ***as shown in the ARRIVE trial (Grobman et al., doi: 10.1056/NEJMoa1800566)*** *but is controversial* ***since in observational studies*** *the procedure, when compared to spontaneous onset of labour, has been associated with harm such as increased need for interventions during labour, uterine rupture, and adverse neonatal outcome.*

*Page 7, line 119: Systematic reviews and randomised clinical trials suggest that induction before 42 gestational weeks may reduce perinatal mortality and severe neonatal morbidity without increasing the frequency of caesarean section, operative vaginal delivery, or postpartum haemorrhage.* ***Furthermore, the ARRIVE trial showed that induction at 39 weeks in low-risk nulliparous women resulted in a significantly lower frequency of caesarean section but it did not change the composite advserse perinatal outcome (Grobman et al., doi: 10.1056/NEJMoa1800566).***

*We have also revised the text in the Discussion section: Page 27, line 554:*

*“Inductions at 39-40 weeks increased. The underlying mechanism for this finding was not investigated in this study and warrants further exploration in the future.* ***However, results from the 35/39 trial in the UK (Walker et al, doi: 10.1056/NEJMoa1509117) and the ARRIVE trial in the USA (Grobman et al., doi: 10.1056/NEJMoa1800566), published in 2016 and 2018, respectively, on elective induction in nulliparous women at 39 weeks of gestation compared with expectant management may have also have had implications for obstetric practice in Sweden and other countries around the world.”***

Methods:
Line 153: When are these outcomes, of birth experience and general health, recorded?

***Answer:*** *Women assessed childbirth experience by a 10-point numeric rating scale before discharge a few days after birth and self-rated health two months after delivery, respectively. This information is given in the Method section, page 9, line 165 and also in the Discussion section, page 25, line 525, but has now also been slightly revised in the Method section.*

*Page 11, line 211 “Additional secondary outcome measures were the maternal experience of childbirth measured* ***before discharge from hospital a few days after birth,*** *and self-rated health two months after delivery, respectively.”*

Line 171: When was the fetus in cephalic presentation - at birth?

***Answer:*** *Yes, at birth. This is specified in the revised manuscript, page 10, line 182.*

Primary outcomes: how were these chosen?  Scalp trauma seems relatively minor (and common at instrumental birth) compared to prolonged neonatal admission.  How were the Apgar score and length of stay cut offs chosen?  References would be helpful.

***Answer:*** *The choice of primary outcomes was based on previous studies, a thorough consideration of the existing knowledge and consensus. For example, the COSIOL (Core outcome sets for induction of labour (Dos Santos et al. Development of a core outcome set for trials on induction of labour: an international multistakeholder Delphi study. BJOG. 2018 Dec;125(13):1673-1680. doi: 10.1111/1471-0528.15397) includes birth trauma and NICU admissions. Length of stay at NICU 4 days or more was chosen because we wanted to* *exclude neonates admitted only for observations at NICU. We chose an Apgar score <4 at five minutes as outcome as an Apgar score <4 at five minutes is associated with an increased risk of long term adverse outcome (Committee Opinion No. 644: The Apgar Score. Obstet Gynecol. 2015 Oct;126(4):e52-e55. doi: 10.1097/AOG.0000000000001108).*

*We have added these references, page 11, line 197 (doi: 10.1111/1471-0528.15397 and line 199 doi: 10.1097/AOG.0000000000001108).*

*And added on page 1, line 199* *“….* ***admission to a neonatal unit for 4 days or more thus excluding neonates admitted only for surveillance at a neonatal unit.”***

Secondary outcomes: line 194, do you mean obstetric anal sphincter injury or full sphincter rupture (ie 3c tear)?  Is level of PPH recorded?

***Answer:*** *Sphincter rupture includes obstetric anal sphincter injury, defined as a third or fourth degree perineal injury involving the external or internal anal sphincter muscles, or both, as defined by the codes O702 and O703 in the Swedish version of the International Classification of Diseases 10th edition.*

*We have clarified this in the Method section, on page 11, line 206 and replaced sphincter injury with* ***“obstetric anal sphincter rupture, defined as a third or fourth degree perineal injury involving the external or internal anal sphincter muscles, or both, as defined by the codes O702 and O703 in the Swedish version of the International Classification of Diseases 10th edition.”***

*Level of PPH is reported in ml, added in the Method section page 11, line 209*

Statistical analysis: Line 200: Why did you not adjust for other maternal comorbidities such as diabetes and hypertension, given that you had the information from ICD-10 codes?

***Answer:*** *Since the rate of type 1 diabetes is low (most women are delivered before 41^+0^ weeks), and unlikely to have changed between the periods, there would be no rationale why it should be adjusted for. Other comorbidities, such as eg. hypertension may be affected by the intervention measured in this investigation (duration of pregnancy). Such factors are mediators, not confounders, and if included in the models, they may seriously bias the results.*

*Furthermore, the ICD 10 codes for gestational hypertension do not differ between hypertension before and after delivery and adjustment for gestational hypertension will therefore not be correct. Lastly, since there was a higher rate of diabetes and hypertension in the second period, adjustment had further strengthened the association found.*

Results: 
This section needs to be reorganised with Table 1 discussed early. I think it's important for the reader's understanding to present very early in the results, the finding that of women over 41 weeks, the proportion going past 42 dropped from 24 to 8 percent (so by 2/3) between the two periods.

***Answer:*** *We have reorganised the Result section and now discuss Table 1 earlier. P-values are deleted in table 1, and the Result section are revised accordingly. Page 15, line 286*

***“Table 1 shows the maternal- and pregnancy characteristics by period of delivery (2017 – 2019 or 2020 – 2023) among pregnancies lasting 41^+0^ weeks or more. The differences in characteristics by period of delivery were small in absolute measures. The induction rate in this cohort increased from 33.7% to 52.4% from period 1 to period 2. Correspondingly, the rate of pregnancies lasting 42^+0^ weeks or more in this population decreased from 24.0% to 7.7%.”***

In general, you need more numbers in the results - at the moment the results only say indicative sentences rather than giving numbers.  E.g. 'A very prominent increase of the induction rate between the two time periods (2017 - 2019 and 2020 - 2023, respectively) was noted among births at 41+1 to 41+6  weeks' - this should have what the induction rate was and what it became in it.

***Answer:*** *We consciously refrained from repeating the numbers already displayed in the tables. However, in the revised version, we have added information on all significant findings (ARR with 95% CI and p-values) displayed in Table 2 and 3, if the editor agrees.*

*We have also added more numbers in the results page 15, line 295: “A very prominent increase of the induction rate between the two time periods (2017 - 2019 and 2020 - 2023, respectively) was noted among births at 41^+1^ to 41^+6^  weeks* ***(from 21% to 55%, p<0.001).*** *Among deliveries at 39^+0^ to 41^+0^ weeks, the induction rate was significantly higher during period 2 than period 1* ***(from 14% to 20%, p<0.001), but among deliveries at 42^+0^ weeks or more, no major change in induction rates between the two study periods was observed (from 77% to 82%, p<0.001).”***

Line 273 onwards about variation should be in a new paragraph.

***Answer:*** *page 15, line 307 onwards about variation, is now in a new paragraph.*

Please include a CI with all reporting of adjusted and unadjusted rate ratios.

***Answer:*** *As previously stated, we tried to avoid reporting double. However, in the revised version we repeat many of the RRs displayed with 95% CI in text.*

Lines 340 onwards: how were these thresholds arrived at?  In general, I think 'tertile' is not a word that's used much - in English we tend to use thirds, I would suggest highest third of … etc.

***Answer:*** *“Tertile” is the noun that is used in statistics. According to Collins English Dictionary, the definition is: “one of three groups with equal frequencies into which a set of data can be divided”. For clarity, if the editor agrees, we would like to keep the word as we aim at the precise definition.*

Line 350: please include RR here and when making statements about RRs.

***Answer:*** *We have added the adjusted RRs and CIs page 19, line 395:”The adjusted RR for peri/neonatal death (period 2 versus period 1) was about the same magnitude among infants born at 41^+0^ to 41^+6^ weeks (****adjusted RR 0.53; 95% CI 0.39-0.72****) as it was for infants born at 42^+0^ weeks or more (****adjusted RR 0.59; 95% CI 0.25-1.40****),* ***p-value for homogeneity=0.823.”***

Discussion:
The authors do not make sufficient reference to the background literature and context - fitting it within the very narrow question of when to put the cut off (41+6 or later) rather than within the broader understanding of when IOL for term, low risk pregnancies should be.  The authors state that the observed increase in induction at 39 weeks needs further investigation, but do not reference the 35/39 trial (published 2016) and ARRIVE trial (published 2018) which both made the offer of induction at 39 weeks much more common across the world.  I would strongly suggest revision with this in mind.

***Answer:*** *As mentioned above we have revised the discussion and added on page 26, line 554: “Inductions at 39-40 weeks increased. The underlying mechanism for this finding was not investigated in this study and warrants further exploration in the future.* ***However, results from 35/39 trial in the UK (Walker et al, doi: 10.1056/NEJMoa1509117) and the ARRIVE trial in the USA (Grobman et al, doi: 10.1056/NEJMoa1800566) published in 2016 and 2018, respectively, which both offered elective induction in nulliparous women at 39 weeks of gestation compared with expectant management may have also have had implications for obstetric clinical practice in Sweden and other countries across the world.”***

Reviewer #3: The authors of the article "Maternal and perinatal outcomes after implementation of a more active management in late-and post-term pregnancies in Sweden: a population-based cohort study" have conducted a significant study. They have demonstrated in a large population-based cohort study that active pregnancy management after the completion of 41 weeks of pregnancy reduces perinatal mortality, as well as neonatal mortality and morbidity. However, they also found an unexplained increase in the cesarean section rate. The research is well-designed, and the methodology is very precisely described, including the statistical analysis used. The results are accurately presented. Due to the extensiveness and additional sensitivity analyses, reading the results is tiring, but the tables and graph complement them perfectly. The discussion summarizes well the main findings of the research and compares them accordingly with other research. They also adequately identify the
shortcomings of the research. The conclusions are consistent with the results.
Although there are already several articles on the subject of induction of labor before and after 41 weeks of pregnancy and there has already been some discussion on the topic at congresses, population-based real-word data research contributes significantly to decisions in clinical practice. The biggest issue, which the research does not really address, is the increase in the rate of caesarean sections. This is a crucial area that requires further investigation. In the discussion, they adequately define this problem with directions for further research. The cited literature is relevant.

***Answer:*** *We thank this reviewer for the over-all positive judgement.*

I did not detect any major flaws in the article. Of the smaller ones, I would mention the title of Table 1, where a reader can understand at first glance that all are excluded:
"Singleton pregnancies, cephalic presentations, elective caesarean sections excluded."

***Answer:*** *We agree. We have adjusted the Table 1 heading. It now says: “****Included were singleton pregnancies in cephalic presentation at birth. Elective caesarean sections were excluded”.***

Any attachments provided with reviews can be seen via the following link: <https://www.editorialmanager.com/pmedicine/l.asp?i=942829&l=YLE5K3GM>
--------------------------------------------------------- ---
General editorial requests:
(Note: not all will apply to your paper, but please check each item carefully)

*       We ask every co-author listed on the manuscript to fill in a contributing author statement, making sure to declare all competing interests. If any of the co-authors have not filled in the statement, we will remind them to do so when the paper is revised. If all statements are not completed in a timely fashion this could hold up the re-review process. If new competing interests are declared later in the revision process, this may also hold up the submission. Should there be a problem getting one of your co-authors to fill in a statement we will be in contact. Please do not add or remove authors without first discussing this with the handling editor. You can see our competing interests policy here: <http://journals.plos.org/plosmedicine/s/competing-interests>.

***Answer:*** *This should be OK*.

*       Please upload any figures associated with your paper as individual TIF or EPS files with 300dpi resolution at resubmission; please read our figure guidelines for more information on our requirements: <http://journals.plos.org/plosmedicine/s/figures>. While revising your submission, please upload your figure files to the PACE digital diagnostic tool, <https://pacev2.apexcovantage.com/>. PACE helps ensure that figures meet PLOS requirements. To use PACE, you must first register as a user. Then, login and navigate to the UPLOAD tab, where you will find detailed instructions on how to use the tool. If you encounter any issues or have any questions when using PACE, please email us at [PLOSMedicine@plos.org](mailto:PLOSMedicine@plos.org).

***Answer:*** *We have checked with PACE and the figures should be OK.*


*       Please ensure that the paper adheres to the PLOS Data Availability Policy (see <http://journals.plos.org/plosmedicine/s/data-availability)>, which requires that all data underlying the study's findings be provided in a repository or as Supporting Information. For data residing with a third party, authors are required to provide instructions with contact information (web or email address) for obtaining the data. Please note that a study author cannot be the contact person for the data. PLOS journals do not allow statements supported by "data not shown" or "unpublished results." For such statements, authors must provide supporting data or cite public sources that include it.

***Answer:*** *We have provided a non-author contact for inquiries about access to data and changed the text on page 29, line 602 to the following:*

***Data availability statement***

*The register data are not publicly available due to privacy protection, including General Data Protection Regulation (GDPR). Access to Swedish register resources is only granted after ethical review by appropriate authorities.* ***Requests should be directed to*** [***registrator@lu.se***](mailto:registrator@lu.se)*. Further information on data access for researchers from the Swedish Pregnancy Register data can be found at* [*registerhallare@graviditetsregistret.se*](mailto:registerhallare@graviditetsregistret.se)*, from the Swedish Neonatal Quality Register at* [*dataservice@snq.se*](mailto:dataservice@snq.se)*, and from Statistics Sweden (https://www.scb.se/vara-tjanster/bestall-dataoch-statistik/).*

*       We expect all researchers with submissions to PLOS in which author-generated code underpins the findings in the manuscript to make all author-generated code available without restrictions upon publication of the work. In cases where code is central to the manuscript, we may require the code to be made available as a condition of publication. Authors are responsible for ensuring that the code is reusable and well documented. Please make any custom code available, either as part of your data deposition or as a supplementary file. Please add a sentence to your data availability statement regarding any code used in the study, e.g. "The code used in the analysis is available from Github [URL] and archived in Zenodo [DOI link]" Please review our guidelines at <https://journals.plos.org/plosmedicine/s/materials-software-and-code-sharing> and ensure that your code is shared in a way that follows best practice and facilitates reproducibility and reuse. Because Github depositions can
be readily changed or deleted, we encourage you to make a permanent DOI'd copy (e.g. in Zenodo) and provide the URL.

***Answer:*** *We have provided a document with the Syntax for the main analyses*

*(S4 Appendix)*

*       Please ensure that the study is reported according to the STROBE guideline and include the completed STROBE checklist as Supporting Information. When completing the checklist, please use section and paragraph numbers, rather than page numbers. Please add the following statement, or similar, to the Methods: "This study is reported as per STROBE guideline (S1 Checklist)."

***Answer:*** *This study will be reported according to RECORD guidelines, please see comment below.*

FORMATTING - GENERAL
*       Abstract: Please structure your abstract using the PLOS Medicine headings (Background, Methods and Findings, Conclusions). Please combine the Methods and Findings sections into one section.

***Answer:*** *This has been checked and should be OK.*

*       At this stage, we ask that you include a short, non-technical Author Summary of your research to make findings accessible to a wide audience that includes both scientists and non-scientists. The Author Summary should immediately follow the Abstract in your revised manuscript. This text is subject to editorial change and should be distinct from the scientific abstract. Ideally each sub-heading should contain 2-3 single sentence, concise bullet points containing the most salient points from your study. In the final bullet point of 'What Do These Findings Mean?', please include the main limitations of the study in non-technical language. Please see our author guidelines for more information: <https://journals.plos.org/plosmedicine/s/revising-your-manuscript#loc-author-summary>.

***Answer:*** *This has been included.*

*       Please express the main results with 95% CIs as well as p values. When reporting p values please report as p<0.001 and where higher as the exact p value p=0.002, for example. Throughout, suggest reporting statistical information as follows to improve clarity for the reader "22% (95% CI [13%,28%]; p</=)". Please be sure to define all numerical values at first use.

***Answer:*** *We have added CIs and p-values as suggested.*

*       Please include page numbers and line numbers in the manuscript file. Use continuous line numbers (do not restart the numbering on each page).

**Answer:** *This should be OK*.


*       Please cite the reference numbers in square brackets. Citations should precede punctuation.

***Answer:*** *This has been corrected.*


FIGURES AND TABLES
*       Please provide titles and legends for all figures and tables (including those in Supporting Information files).

***Answer:*** *This has been provided.*

*       Please define all abbreviations used in each figure/table (including those in Supporting Information files).

***Answer:*** *Abbreviations have been defined.*

*       Please consider avoiding the use of red and green in order to make your figure more accessible to those with color blindness.

***Answer:*** *Figure 2 has been revised.*


SUPPLEMENTARY MATERIAL
*       Please note that supplementary material will be posted as supplied by the authors. Therefore, please amend it according to the relevant comments outlined here.

*       Please cite your Supporting Information as outlined here: <https://journals.plos.org/plosmedicine/s/supporting-information>

***Answer:*** *This has been cited as outlined.*


REFERENCES
*       PLOS uses the numbered citation (citation-sequence) method and first six authors, et al.

***Answer:*** *This has been revised according to this instructions.*

*       Please ensure that journal name abbreviations match those found in the National Center for Biotechnology Information (NCBI) databases (<http://www.ncbi.nlm.nih.gov/nlmcatalog/journals>), and are appropriately formatted and capitalised.

***Answer:*** *This has been revised according to these instructions.*


*       Where website addresses are cited, please include the complete URL and specify the date of access (e.g. [accessed: 12/06/2023]).

***Answer:*** *This has been checked and revised according to these instructions.*

*       Please also see <https://journals.plos.org/plosmedicine/s/submission-guidelines#loc-references> for further details on reference formatting.

***Answer:*** *This has been checked and revised according to these instructions.*


OBSERVATIONAL STUDIES

*       Abstract: Please include the study design, population and setting, number of participants, years during which the study took place (enrollment and follow up), length of follow up, and main outcome measures.

***Answer:*** *We have added cohort in Methods and Findings page 4, line 63: “…nationwide, register based* ***cohort*** *study.”*

*       [FOR POPULATION HEALTH/REGISTRY STUDIES] Please ensure that the study is reported according to the RECORD guideline (available from [[https://www.record-statement.org)]https://www.record-statement.org](https://www.record-statement.org)]https/www.record-statement.org)) and include the completed checklist as Supporting Information. Please add the following statement, or similar, to the Methods: "This study is reported as per the Reporting of Studies Conducted using Observational Routinely-Collected Data (RECORD) guideline (S1 Checklist)." When completing the checklist, please use section and paragraph numbers, rather than page numbers.

***Answer:*** *We have added in the Method section, page 9, line 148:* ***“This study is***

***reported as per the Reporting of Studies Conducted using Observational Routinely-Collected Data (RECORD) guideline (S1 Checklist)****." We have added the RECORD Checklist as a Supplementary file (S1 Appendix).*

*       For all observational studies, in the manuscript text, please indicate: (1) thespecific hypotheses you intended to test, (2) the analytical methods by which you planned to test them, (3) the analyses you actually performed, and (4) when reported analyses differ from those that were planned, transparent explanations for differences that affect the reliability of the study's results. If a reported analysis was performed based on an interesting but unanticipated pattern in the data, please be clear that the analysis was data driven.

***Answer:*** *1. We have added the specific hypothesis we intended to test in the last section of the Introduction, page 8, line 142:* ***“The hypothesis was that early induction improved perinatal outcome”.***

*2. the analytical methods we planned and 3. the analyses we performed are*

*presented in the Statistical methods. 4. No reported analyses different from those*

*that were planned are included.*

*       Please state in the Methods section whether the study had a prospective protocol or analysis plan. If a prospective analysis plan (from your funding proposal, IRB or other ethics committee submission, study protocol, or other planning document written before analyzing the data) was used in designing the study, please include the relevant document(s) with your revised manuscript as a Supporting Information file to be published alongside your study and cite it in the Methods section. A legend for this file should be included at the end of your manuscript. If no such document exists, please make sure that the Methods section transparently describes when analyses were planned, and when/why any data-driven changes to analyses took place. Changes in the analysis, including those made in response to peer review comments, should be identified as such in the Methods section of the paper, with rationale.

***Answer:*** *The study had ethical permission and a study protocol with a prospective analysis plan. These documents (in Swedish) are included as Supporting information. No data driven changes to analyses took place. We have added in the Method section, page11, line 214:* ***“The analysis followed the ethical approval (S2 Appendix) and the prospective study protocol detailing the planned analysis (S3 Appendix). Changes from the planned protocol as response to peer review comments were 1. multiple imputation used to replace missing information on BMI or smoking for the main outcomes and 2. the additional analysis to control for possible difference in hospital practice.”***
