# Supplementary material for: Maternal and perinatal outcomes after implementation of a more active management in late- and postterm pregnancies in Sweden: A population-based cohort study
Source: PLoS Med. 2025 Jan 16;22(1):e1004504. doi: 10.1371/journal.pmed.1004504 (PMC11737695; doi:10.1371/journal.pmed.1004504)
Supplement: Attachment — Submitted filename: Answer Comments from Reviewers 241111.docx [file pmed.1004504.s008.docx]

Comments from Reviewers:

Reviewer #1: The authors have addressed my points.

Just to clear up a small misunderstanding about titles not in English. I know they do not have an official translation but the authors could supply their version or a comment so that people know what the content might be to save them going to the trouble of downloading it, putting it through a machine translation and then finding out it was irrelevant to their interests.

Michael Dewey

***Answer:*** *Thank you for this clarification. We have provided an explanation to the text in Norwegian and Swedish for the following three references:*

*23.https://www.helsebiblioteket.no/innhold/nasjonal-faglig-retningslinje/fodselsomsorgen-nasjonal-faglig-retningslinje#svangerskap-passert-termin-gravide-som-har-passert-termin-bor-senest-igangsettes-for-fodsel-uke-420 [accessed September 26, 2024]. Norwegian national guidelines for management of late term pregnancies.*

*24. https://vardpersonal.1177.se/globalassets/nkk/nationell/media/dokument/*

*kunskapsstod/vardriktlinjer/riktlinje-for-handlaggning-i-graviditetsvecka-41.pdf [accessed September 26, 2024]. Swedish national guidelines for management of late term pregnancies.*

*48. https://www.sfog.se/start/kunskapsstoed/obstetrik/preeklampsi [accessed September 26, 2024]. Guidelines from the Swedish Society of Obstetrics and Gynecology*

*for management of hypertensive disorders in pregnancy.*

Reviewer #2: Responses to my queries are adequate, thank you.

***Answer:*** *Thank you!*


Any attachments provided with reviews can be seen via the following link:


------------------------------------------------------------
Requests from Editors:

CODE AVAILABILITY

Thank you for providing a document with the syntax for the main analyses (S4 Appendix). We kindly ask you to include a sentence about the availability of the code in the data availability statement in the online submission form.

**Answer:** *We have included the following sentence in the data availability statement:
Page 27, line 584. Syntax for the main analyses is shown in S1 Supporting information.*

ABSTRACT

1) l.72: Please include the important dependent variables that are adjusted for in the analyses.

***Answer:*** *We have added the following information in the Abstract, page 4, line 72*

*Relative risks (RR) with 95% confidence intervals (CI) for binary outcomes period 2 versus period 1 were computed using modified Poisson regression analyses with* *adjustments for maternal age, parity, body mass index, smoking, and educational level.*

2) ll.74-75: When reporting means, please provide the standard deviation.

***Answer:*** *We have added standard deviation (SD), page 4-5, line 74-75.*

*Mean (standard deviation) gestational age at birth decreased from 290.7 (2.9) days to 289.6 (2.3) days.*

*SD is also added on page 16, line 317*

*Among pregnancies lasting 41^+0^ or more, the mean gestational age at birth decreased marginally, from 290.7 (2.9) days in period 1 to 289.6 (2.3) in period 2 (difference 1.13 days, 95% CI [1.10, 1.16]).*

3) l.76: Please specify what the number 1000 refers to, at least the first time you report it.

***Answer:*** *We have added this information page 5, line 76-77.*

*Infants born during period 2 were at lower risk of peri/neonatal death compared to infants born during period 1; 0.9/1000 versus 1.7/1000 born infants.*

4) We suggest reporting statistical information as follows to improve clarity for the reader "22% (95% CI [13%,28%]; p</=)". When reporting 95% CIs please separate upper and lower bounds with commas instead of hyphens as the latter can be confused with reporting of negative values. For example, lines 76-77: “(adjusted RR 0.52; 95% CI [0.38,0.69]; p<0.001)”

***Answer:*** *We have corrected this throughout the manuscript.*

5) In the last sentence of the Abstract Methods and Findings section, please describe the main limitation(s) of the study's methodology.

***Answer:*** *We have added the following sentence, page 5, line 83-84.*

*One limitation of the study is that we had no data on to what extent monitoring of fetal health was performed.*

6) If you agree, we suggest adding a sentence about the implications of the study in the Abstract Conclusion, as is done in the Author Summary under 'What do these findings mean?’.

***Answer:*** *We agree and added a sentence according to your suggestion about the implications of the study in the Abstract Conclusion, page 5, line 88-92. However, with this sentence the abstract exceeded the word limit (509 words).*

*Women with pregnancies advancing towards 41 gestational weeks should be given balanced information on the benefits and risks of induction of labour at 41 weeks compared to expectant management until 42 weeks and be offered induction of labour at 41 weeks or active surveillance of pregnancies from 41 weeks in order to decrease peri/neonatal mortality.*

AUTHOR SUMMARY

Thank you for providing the Author Summary. Please revise with regard to formatting. Each sub-heading (please note the question format) should contain 2-3 single sentence, concise bullet points containing the most salient points from your study. In the final bullet point of 'What Do These Findings Mean?', please include the main limitations of the study in non-technical language.

***Answer:*** *We have revised the author summary with regard to formatting (bullet points), and added the main limitation of the study in the final bullet point of 'What Do These Findings Mean? Page 6, line 115.*

***Why was this study done***

- *Rare but severe outcomes such as stillbirth, perinatal mortality, and severe neonatal and maternal morbidity are more common among late-and postterm pregnancies than among full term pregnancies.*
- *Randomised clinical trials have shown decreased perinatal mortality by labour induction of late-and postterm pregnancies while real-world observational studies have shown conflicting results after such an intervention.*

***What did the researchers do and find***

- *We have performed a register based national study, which confirms earlier randomised clinical trials, demonstrating decreased rates of peri/neonatal mortality and a composite outcome of peri/neonatal mortality and severe neonatal morbidity including birth trauma after offering pregnant women induction of labour or an individual plan from 41 instead of 42 gestational weeks.*
- *An increase in caesarean sections was also observed.*

***What do these findings mean***

- *Women with pregnancies advancing towards 41 gestational weeks should be given balanced information on the benefits and risks of induction of labour at 41 weeks compared to expectant management until 42 weeks and be offered induction of labour at 41 weeks or active surveillance of pregnancies from 41 weeks in order to decrease peri/neonatal mortality.*
- *One limitation of the study is that we had no data on to what extent monitoring of fetal health was performed.*

INTRODUCTION

1) l.107: Please note that ‘(40+6 weeks)’ should have two right parentheses.

***Answer:*** *Corrected*

2) l.129: Please define ‘WHO’ at first use.

***Answer:*** *We have defined WHO as World Health Organization at first use, page 8, line 144*

METHODS AND RESULTS

1) Table 1: Please define ‘Nordic country’ below the table.

***Answer:*** *We have added a definition of Nordic countries below Table 1:*

*Nordic countries include Denmark, Finland, Iceland, Norway, and Sweden.*

2) Table 1: For ‘child characteristics’, we suggest changing the first one to ‘Sex, Male’. Please note that the terms gender and sex are not interchangeable (as discussed in <https://www.who.int/health-topics/gender#tab=tab_1> ); please use the appropriate term.

***Answer:*** *We have corrected to ‘Sex, male’ in Table 1.*

3) Table 2: Please define ‘CI’ below the table. Please see the comments regarding reporting of statistical information (under Abstract) and revise accordingly.

***Answer:*** *We have defined ‘CI’ below the table and revised reporting of statistical information in the tables.*

4) Table 3: Please define ‘CI’ and ‘RR’ below the table.

***Answer:*** *We have defined ‘CI’ below the table and ‘RR’ in the heading as in Table 1.*

5) Table 4: Please define ‘CI’ and ‘RR’ below the table. Please add a unit for BMI. Please define ‘Nordic country’ below the table. Please add a brief explanation about the hospital induction change tertile as tables should be self-explanatory on a stand-alone basis.

***Answer:*** *We have defined ‘CI’ below the table 4 and ‘RR’ in the heading as in Table 1.*

*We have added a unit and explanation for BMI and defined the Nordic countries.*

*BMI = kg/m^2^; kg is a person's weight in kilograms and m^2^ is height in metres squared.*

*Nordic countries include Denmark, Finland, Iceland, Norway, and Sweden.*

*We have added the following explanation about the hospital induction change tertile:*

*The Swedish delivery hospitals were categorized into three tertiles according to the magnitude of the induction rate change among pregnancies 41+0 weeks or more between the time periods and the relative risks (period 2 versus period 1) for outcomes were individually computed for each hospital tertile category.*

6) Figure 1: Please define ‘CS’. Please note that words in the image are underlined in red, probably by the spell check function.

***Answer:*** *Figure 1 is replaced and ‘CS’ is defined in Figure 1.*

7) ll.388-391: Apologies if I missed it, but are these results presented anywhere in a supplementary table? Please refer to the relevant table or provide such a table.

***Answer:*** *We have added data with results from sensitivity analyses excluding births*

*during 2017 or infants with significant congenital malformations with these results*

*presented in a new table, S2 Table in S1 Supporting Information.*

DISCUSSION

1) Please remove any subheadings from the discussion section, including the ‘conclusion’ subheading.

***Answer:*** *We have removed the subheading from the discussion including the ‘conclusion’ subheading.*

2) l.400: “per thousand”? – please specify.

***Answer:*** *We have specified this, page 20, line 419:*

*“..from 1.7 per thousand to 0.9 per thousand born infants in absolute values”.*

SUPPLEMENTARY MATERIAL

In the published article, supporting information files are accessed only through a hyperlink attached to the captions. For this reason, you must list captions at the end of your manuscript file. You may include a caption within the supporting information file itself, as long as that caption is also provided in the manuscript file. Do not submit a separate caption file.

When SI files are contained with a single file:
Please label the file as ‘S1 Supporting Information’.
Please apply alphabetical labelling to each table and figure contained within the S1 file. For example, ‘Fig A’ to ‘Fig Z’ and ‘Table A’ to ‘Table Z’.
Plain text does not need to be labelled and can just be given a title as necessary. For example, ‘Statistical Analysis Plan’.
Please cite tables/figures as ‘Fig A in S1 Supporting Information’ and/or ‘Table A in S1 Supporting Information’, for example.
Please cite plain text as, ‘Statistical Analysis Plan in S1 Supporting Information’, for example.

When SI files are uploaded as separate files:
Please label tables as ‘S1 Table’ (so on) and figures as ‘S1 Fig’ (and so on).
Any additional documents (protocols/analysis plans etc.) can be labelled as ‘S1 Protocol’, for example. Please cite items as exactly as labelled.

***Answer:*** *Supporting information has been revised. The supporting information files are uploaded as separate files (S1 RECORD Checklist, S1 Ethical permission, S1 Protocol, and S1 Syntax Main Analysis, S1Table, and S2 Table) and cited in text as*

*S1 RECORD Checklist, S1 Ethical permission, S1 Protocol, S1 Syntax Main Analysis, S1 Table, and S2 Table in S1 Supporting Information.*

*Captions are listed at the end in the manuscript file, page 28, line 607-615.*

SOCIAL MEDIA

To help us extend the reach of your research, please provide any X (formerly known as Twitter) handle(s) that would be appropriate to tag, including your own, your co-authors’, your institution, funder, or lab. Please enter in the submission form any handles you wish to be included when we post about this paper.

***Answer:*** *We have no such information.*


------------------------------------------------------------
General Editorial Requests

1) We ask every co-author listed on the manuscript to fill in a contributing author statement. If any of the co-authors have not filled in the statement, we will remind them to do so when the paper is revised. If all statements are not completed in a timely fashion this could hold up the re-review process. Should there be a problem getting one of your co-authors to fill in a statement we will be in contact. YOU MUST NOT ADD OR REMOVE AUTHORS UNLESS YOU HAVE ALERTED THE EDITOR HANDLING THE MANUSCRIPT TO THE CHANGE AND THEY SPECIFICALLY HAVE AGREED TO IT.

***Answer:*** *All authors have filled in a contributing author statement.*

2) Please ensure that the paper adheres to the PLOS Data Availability Policy (see <http://journals.plos.org/plosmedicine/s/data-availability)>, which requires that all data underlying the study's findings be provided in a repository or as Supporting Information. For data residing with a third party, authors are required to provide instructions with contact information for obtaining the data. PLOS journals do not allow statements supported by "data not shown" or "unpublished results." For such statements, authors must provide supporting data or cite public sources that include it.

***Answer:*** *Contact information for obtaining data is provided.*

3) Please review your reference list to ensure that it is complete and correct. If you have cited papers that have been retracted, please include the rationale for doing so in the manuscript text, or remove these references and replace them with relevant current references. Any changes to the reference list should be mentioned in the rebuttal letter that accompanies your revised manuscript.

***Answer:*** *No changes have been made in the reference list, and we hope the list is correct and complete.*

__________________________________________________
In compliance with data protection regulations, you may request that we remove your personal registration details at any time.  (Use the following URL: <https://www.editorialmanager.com/pmedicine/login.asp?a=r)>. Please contact the publication office if you have any questions.
